# Surfaceome interrogation using an RNA-seq approach highlights leukemia initiating cell biomarkers in an *LMO2* T cell transgenic model

**DOI:** 10.1038/s41598-019-42214-w

**Published:** 2019-04-08

**Authors:** Helio Pais, Katia Ruggero, Jing Zhang, Osama Al-Assar, Nicolas Bery, Ravneet Bhuller, Victoria Weston, Pamela R. Kearns, Cristina Mecucci, Ami Miller, Terence H. Rabbitts

**Affiliations:** 1 MRC Weatherall Institute of Molecular Medicine, MRC Molecular Haematology Unit, University of Oxford, Oxford, OX3 9DS UK; 2 Present Address: Trivago GmbH, Bennigsen-Platz 1, 40474 Düsseldorf, Germany; 3 Present Address: Catalan Institute of Oncology, Bellvitge Institute for Biomedical Research (IDIBELL), Gran Via de L’Hospitalet, 199, Barcelona, Spain; 4 Present Address: The Wellcome Centre for Human Genetics, Roosevelt Drive, Oxford, OX3 7BN UK; 5 Institute of Cancer and Genomic Sciences, College of Medical and Dental Sciences, University of Birmingham, Vincent Drive, Edgbaston, B15 2TT UK; 6 University Hospital A.O., Perugi, Italy

## Abstract

The surfaceome is critical because surface proteins provide a gateway for internal signals and transfer of molecules into cells, and surfaceome differences can influence therapy response. We have used a surfaceome analysis method, based on comparing RNA-seq data between normal and abnormal cells (Surfaceome DataBase Mining or Surfaceome DBM), to identify sets of upregulated cell surface protein mRNAs in an LMO2-mediated T-ALL mouse model and corroborated by protein detection using antibodies. In this model the leukemia initiating cells (LICs) comprise pre-leukaemic, differentiation inhibited thymocytes allowing us to provide a profile of the LIC surfaceome in which GPR56, CD53 and CD59a are co-expressed with CD25. Implementation of cell surface interaction assays demonstrates fluid interaction of surface proteins and CD25 is only internalized when co-localized with other proteins. The Surfaceome DBM approach to analyse cancer cell surfaceomes is a way to find targetable surface biomarkers for clinical conditions where RNA-seq data from normal and abnormal cell are available.

## Introduction

The spectrum of cell surface proteins (the surfaceome) is critical to normal and disease biology including normal embryonic development and occurrence of abnormal cells in the origin of diseases. Simple methods are needed to interrogate surfaceomes and in particular where low numbers of cells are available. The next generation deep sequencing of whole RNA-seq transcriptomes is a starting point that allows the whole spectrum of surfaceome mRNAs to be identified in cells and subtractive analysis allowing comparison of two or more cell types^1^. Proteomic methods to define the protein repertoire of cells have increased sensitivity in recent years with major improvements in application of mass spectrometry (MS)^2,3^. However, evaluation of the complete proteome of small numbers of cells remains a major challenge^4^. RNA-seq deep sequencing will, however, reveal even the lowest abundance mRNAs and, followed by specific protein analysis, can define the whole proteome, including those proteins that are located on the cell surface. We have recently applied the use of RNA-seq for defining cell surface expression^1^ by developing a surfaceome database that can be used to define cell-specific mRNA expression. The surfaceome database was constructed using publicly available data on genes to catalogue all those known to (or likely to) encode cell surface or secreted proteins (www.rdm.ox.ac.uk/research/rabbitts-group). This approach is not only applicable to new RNA-seq data but also to meta-analysis of published data where only RNA-seq data are available. Furthermore, RNA-seq is applicable to situations where very few cells are available and even at the level of single cells^5^.

We have applied the RNA-seq surfaceome method to the analysis of LICs from an LMO2-dependent transgenic mouse model of T-ALL where the pre-symptomatic stages are characterized by immature thymocyte accumulation^6^ and in which these immature thymocytes behave as leukemia stem cells since they self-renew and can be serially transplanted^7^. The thymocyte populations suffer a differentiation block at the stage of CD4; CD8-double negative cells^6,8^ and specifically at the DN2/DN3 stage (CD4−; CD8−; CD44+;CD25+ or CD4−; CD8−; CD44−; CD25+)^7^. Thus the LIC target population in this disease is the CD25-expressing DN2 (CD4−; CD8−; CD44+; CD25+) and DN3 (CD4−; CD8−; CD44−; CD25+) population of immature thymocytes. Since there is no readily accessible pre-leukemic phase of human T-ALL and there is no high-risk group where pre-cancer might be studied, this mouse model is an important resource for studying T-ALL LICs by providing insight into the pre-leukemic phase of T-ALL pathogenesis. LMO2 is expressed in more than 50% of human T-ALL^9,10^ and LMO2 mRNA was found in 42% of pediatric/adult T cell leukemia (as judged from RNA-seq, with Fragments per Kilobase of transcript per million mapped reads (FPKM) values above 4)^11^, while it is not expressed in normal human/mouse peripheral T cells^9,10,12^, and the protein has no known functional role at this stage of T cell development^13^. Further corroboration of the selectivity of normal cell LMO2 expression was obtained by examining RNA-seq data for human CD4, CD8 double negative thymocytes (defined as CD34+ CD7+ CD1a+ CD4− CD8−; Thy3^11^) where there is no LMO2 mRNA expression, whereas thymocyte progenitors (Thy1, Thy2) have LMO2 mRNA (Supplementary Table S1A). The human thymocyte subsets CD4+; CD8+ (Thy4) or CD3+ CD4+ CD8− (Thy5) or CD3+ CD4− CD8+ (Thy6) have no LMO2 mRNA. These normal human thymocyte data are consistent with our data from fractionated wild type mouse DN thymocytes (Supplementary Table S1B).

We have applied our surfaceome interrogation approach to our LMO2 mouse model by using fractionated immature thymocytes for RNA-seq analysis and generating surfaceome data of the transgenic compared to wild type mouse thymocytes. We have also compared these with data obtained from a set of human primary overt T-ALL samples. This analysis has shown surface markers that are highly expressed in the transgenic LIC cells of the pre-leukemic phase compared to wild type counterparts and combinations of expressed surface markers that could represent multiple targets for therapy, namely CD25, GPR56, CD53, CD59a. Further, employing new methods to assess if surface proteins can co-associate and internalize, we found that there is fluid interaction of surface proteins and CD25 can associate with any of GPR56, CD53 and CD59a at the cell surface, it will only internalize with antibody-cross linking when associated with another surfaceome protein. Our work shows that LMO2-induced T-ALL LICs do not express any single surface marker that can be employed for fractionation of the LIC population or for therapeutics against that population. Fractionation of LIC populations will need combinations of markers (bi- or tri-specific ligands, most notably that include anti-CD25) and, in turn, therapies that are targeting the T-ALL LICs should also ideally employ bi- or tri-specific ligands. Further, the delivery of macrodrugs, such as intracellular antibody fragments, within therapeutic vehicles such as nanoparticles, can exploit our findings of the surfaceome phenotype of the LICs.

## Results

### Using RNA-seq to define immature thymocyte surfaceome mRNAs in *Lck-LMO2* mice

As a means to define LIC surface markers, the cell surface protein expression profile of DN3 cells from *Lmo2* transgenic were analyzed using RNA-seq as a surrogate for protein expression. The RNA from sub-fractions of mouse thymocytes were prepared as described in Methods. The yields of cell numbers from *Lck-Lmo2* and wild type mouse flow cytometry are shown in Supplementary Fig. S1. RNA-seq data were obtained and a comparison was made of the mRNAs encoding cell surface proteins (the surfaceomes) using our in-house database comprising genes that encode surface and secreted proteins^1^. The surfaceome data are displayed in Supplementary Table S2, comprising a list of mRNAs for each DN subset in the surfaceome database that allowed a differential comparison of the DN sub-populations in *LMO2* expressing and wild type thymocytes.

**Figure 1:**
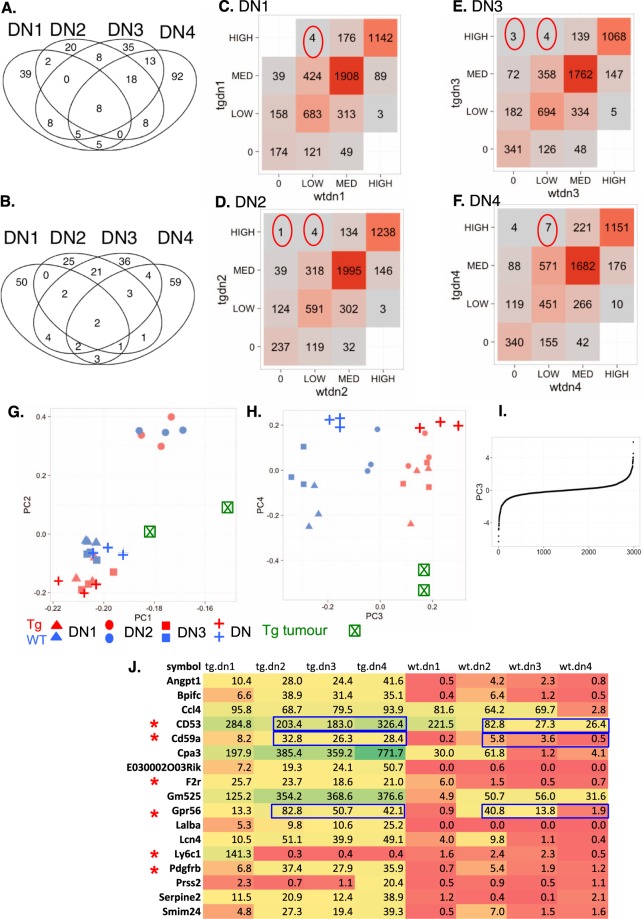
Comparison of surfaceome mRNA expression levels between *Lck-Lmo2* transgenic and wild type purified thymocytes from the four DN stages. The four CD4-CD8- DN thymocyte subsets were purified from 20-week *Lck-Lmo2* transgenic and wild type (WT) mice. RNA-seq data were converted to TPM to analyze the surfaceome database^1^. (**A**,**B**) Venn diagrams of the differentially expressed mRNAs (**A** up-regulated; **B** down-regulated) from *Lck-Lmo2* DN subsets compared to WT, considering that an mRNA is up-regulated if average TPM is greater than 1 in the *Lck-Lmo2* samples and there is more than 10-fold change between *Lck-Lmo2* and WT (only 8 are differentially expressed in all 4 stages). The corresponding mRNAs are included in Supplementary Table S3. (**C**–**F**) Summarize expression comparisons between *Lck-Lmo2* transgenic and WT mice surfaceome for each DN stage. In each the expression profile is summarized by placing the mRNAs into 4 expression classes (total of 16 possible tiles). (**G**–**I**) Principal Component (PC) analysis. Expression levels were transformed by a logarithmic function before calculation of PCs. (**G**) Sample scatter plot on the first and second PCs. The second PC clearly separates DN1 samples from other samples (**H**) sample scatter plot on the third and fourth PCs. Third PC separates transgenic and wild type samples while the third PC separates DN1-DN2-DN3 samples in wild type. (**I**) Sorted mRNA weights used to build the third PC. The x-axis corresponds to the sort order of mRNAs by the third PC weight and the y-axis to the mRNA weights in the third PC. The majority of mRNAs has a near null contribution to the third PC; only a small proportion of mRNAs contributes significantly to this PC. (**J**) A heat diagram comparing TPM values of surfaceome comparators from DN1-DN4 of the *Lck-Lmo2* thymocytes (tg,dn1 etc.) with wild-type (wt,dn1 etc.). Known surfaceome protein-encoding mRNAs are marker with the red asterisks and the values for GPR56, CD53 and CD59a are highlighted in blue boxes.

The Venn diagrams (Fig. 1A,B) display the numbers of surface protein-encoding mRNAs that are up-regulated or down-regulated comparing *Lck-LMO2* with wild type mice. The mRNAs found in the DN T cell subsets are listed in Supplementary Table S3 according to whether an mRNA is only seen in one of the DN subsets or of mixed occurrence (a full list is given in Supplementary Table S4). In further analysis, we placed mRNAs into four distinct expression level classes: 0 (expression level <0.1 TPM), low (between 0.1 and 1 TPM), medium (between 1 and 10 TPM) and high (>10 TPM). The number of transcripts that fall inside each bin is shown in Fig. 1C–F. This suggests that the majority of cell surface protein encoding mRNAs have similar expression levels in LMO2-expressing and wild type thymocytes. The correlation of expression levels is higher for samples from the same stage and different genotype than samples from the same genotype but different stage (Supplementary Fig. S2). Moreover, for each of the four DN stages we observe small numbers of transcripts with large differences in expression levels between *Lck-LMO2* and wild type mouse surfaceome mRNAs.

The overall relationship of the surfaceome mRNAs in the four DN thymocyte populations was examined using Principle Component Analysis (Fig. 1G–I). The first two principal components clearly separate the DN1 and DN2 samples (both *Lck-Lmo2* and wild type) from the rest (Fig. 1G). The third principal component separates *Lck-Lmo2* samples (all stages) from wild type samples (also all stages, Fig. 1H). For wild type samples (but not *Lck-Lmo2* samples) the third principal component also separates DN1 samples from the remaining three stages. This is presumably a reflection of the differentiation block that occurs in *Lck-Lmo2* DN2/DN3 stages. An inspection of the distribution of weights that define the third principal component (Fig. 1I) further reveals that it is dominated by only a small number of mRNAs. This pattern suggests that the differentiation block in *Lck-Lmo2* DN3 cells corresponds to small changes in the mRNA expression cell surface profiles.

**Figure 2:**
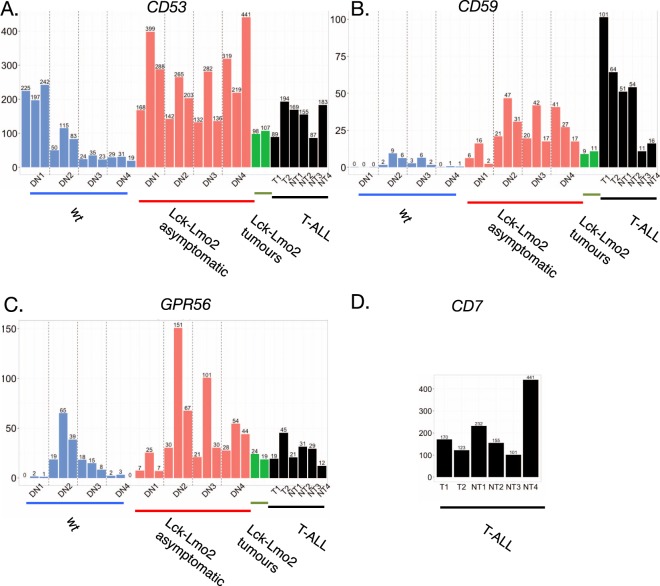
Expression levels of candidate target surface proteins in immature thymocytes and mouse or human T cell neoplasias. The RPKM values for four surface markers (y axis) for the triplicate samples of DN1, 2, 3 and 4 from wild-type (blue) or asymptomatic *Lck-Lmo2* (red) compared to two thymocyte tumors in *Lck-Lmo2* mice (green) or human primary T-ALL samples (black; TN1, 2 and NT1-4) (Supplementary Table S7). Panel A: *CD53* values. Panel B: *CD59a* values. Panel C: *GPR56* values; Panel D: *CD7* values (NB: *Cd7* could not be computed for mouse mRNA as the mouse equivalent of *CD7* is equivocal).

Cell surface proteins in the *Lck-Lmo2* DN3 LICs were assessed using RNA-seq analysis and protein validation to identify markers of these cells for targeting using average TPM higher than 20 and a large expression difference between the transgenic and wild-type samples (Supplementary Table S5). There are six mRNAs coding for membrane-associated proteins that appear in the heat-map of Fig. 1J, of which GPR56, CD53, CD59a, F2R and platelet derived growth factor beta (PDGRFB) have higher TPM values compared to the wild type DN cells. PDGRFB is not restricted to T cell lineages and F2R mRNA expression levels are constant in all DN stages. The expression level of mRNAs encoding GPR56, CD53 and CD59a are present in the DN2, DN3 and DN4 cells of *Lck-LMO2* transgenic at more than 5–10 times compared to wild type (Fig. 1J, Supplementary Table S3C) and expression is sustained through these stages in the *Lck-Lmo2* transgenic mice. From these analyses, the mRNA expression of GPR56, CD53, CD59a is the most significant for cell surface protein expression in the DN3 LIC target cells of the *Lck-Lmo2* mice. Histograms of the TPM for GPR56, CD53 and CD59a from the purified, separated *Lck-Lmo2* and wild type DN cells are shown in Fig. 2A–C. Comparing RNA-seq data of these three markers in our human T-ALL panel with the well-characterized CD7 T cell marker^14^ showed that all are highly expressed at the mRNA level (Fig. 2A–D).

### Flow cytometry confirms mRNA-protein correlation in LMO2-dependent mouse thymocytes

**Figure 3:**
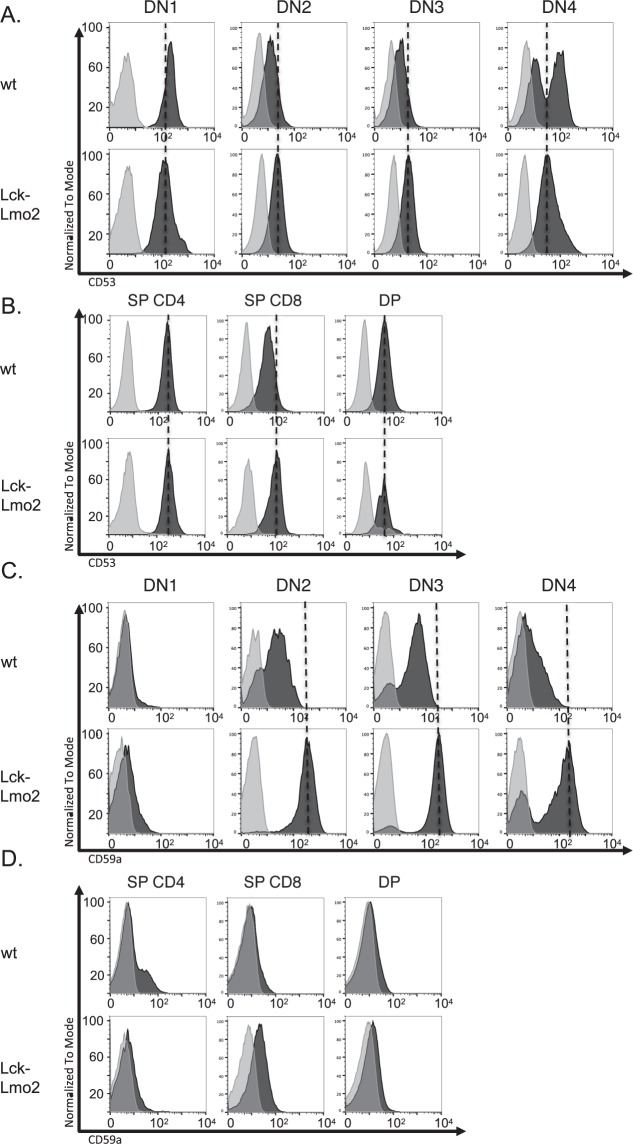
Flow cytometry analysis of CD53 and CD59a expression on mouse *Lck-Lmo2* DN thymocytes. Thymus cells were prepared from asymptomatic *Lck-Lmo2* mice or equivalent age wild type mice and expression of CD53 (panel A; wild type mice 15 weeks old; *Lck-Lmo2* 21 weeks old) or CD59a (panel B; wild type 16 weeks old, *Lck-Lmo2* 19 weeks old) was profiled in the CD4-/CD8- DN subsets (The histogram shows the results for DN cells on stained cells (black) and cells stained with isotype control (light grey). The following fluorescent antibodies were used: CD90-V450, CD4-PE-cy7, CD8-PE, CD44-V500, CD25-APC, CD53-BB515. The x-axis shows fluorescence on a log scale. Panels C,D show the flow cytometry data for the same mice comparing the expression of CD53 (panel C) and CD59a (panel D) in single CD4 positive (SP), single CD8 positive and CD4/CD8 double positive (DP) thymocytes. NB: No suitable antibody is available binding to mouse GPR56.

The surface protein levels were assessed using fluorescent antibodies in flow cytometry with mouse thymocytes. Suitable antibodies are only available for CD53 and CD59a in mouse and we were able to confirm protein expression in asymptomatic, immature CD25+ T cells in mice for those two markers (i.e. DN2, DN3 cells). Flow cytometry shows expression of CD53 on all mouse DN subsets (Fig. 3A) as well as the CD4+ CD8+ double positive and single positive thymocytes (Fig. 3B). We further evaluated CD53 expression in DN T cells, comparing *Lck-Lmo2* transgenic and wild type mice, and comparing *Lck-Lmo2* mice at 15 and 21 weeks (Supplementary Fig. S3). A statistically significant variance was found in the DN2/3 subset between *Lck-Lmo2* and wild type (p value, 0.0238) whereas DN4 from wild type mice displayed two populations of CD53 expressing cells (shown by high and low fluorescence) whilst the *Lck-Lmo2* DN4 cells did not have the CD53-low cells. CD59a expression begins at the DN1 stage of the *Lck-Lmo2* mice, and is highly expressed through to DN4 compared with wild type mice (Fig. 3C). CD59a persists in the DN4 cells of older *Lck-Lmo2* mice (Supplementary Fig. S4). While wild type DN2, DN3 and DN4 cells express surface CD59a, the levels are an order of magnitude higher in the *Lck-Lmo2* mice, especially evident in some mice that have dual high and a moderate-expressing population of cells (Supplementary Fig. S4). Both *Lck-Lmo2* transgenic and wild type mice express low CD59a on DP and SP cells (Fig. 3D). The LICs in the asymptomatic, pre-leukemic phase in the mice (CD25-expressing DN3 T cells) co-express CD53 and CD59a proteins and mRNA for the GPR56 marker. The finding of GPR56 expression is in concordance with recent bioinformatics analysis of LSC and ‘stemness’ in AML^15^.

### RNA-seq surfaceome biomarkers are present on overt human T-ALL

We have examined a panel of fifteen primary human T-ALL samples (with and without *LMO2* translocations) to determine if the markers found in the mouse DN LICs are expressed. RNA-seq was performed with six of these primary human T-ALL mRNA samples and compared to RNA-seq from two *Lck-Lmo2* transgenic mouse tumors. The TPM values for *LMO2* mRNA in the six human T-ALL samples shows a correlation of high expression with the *LMO2* chromosomal translocation gene compared to the non-translocated T-ALL (Supplementary Table S6). Western blot analysis of LMO2 protein expression was performed using samples from which sufficient material was available, and all showed expression of LMO2 protein (Supplementary Fig. S5). The RNA transcriptomes for all the tumor samples showed the three markers GPR56, CD53 and CD59a are expressed as mRNA, together with low expression of CD25 (Supplementary Table S7). The TPM values for the surface biomarkers in our human T-ALL panel are summarized in Supplementary Table S8A.

**Figure 4:**
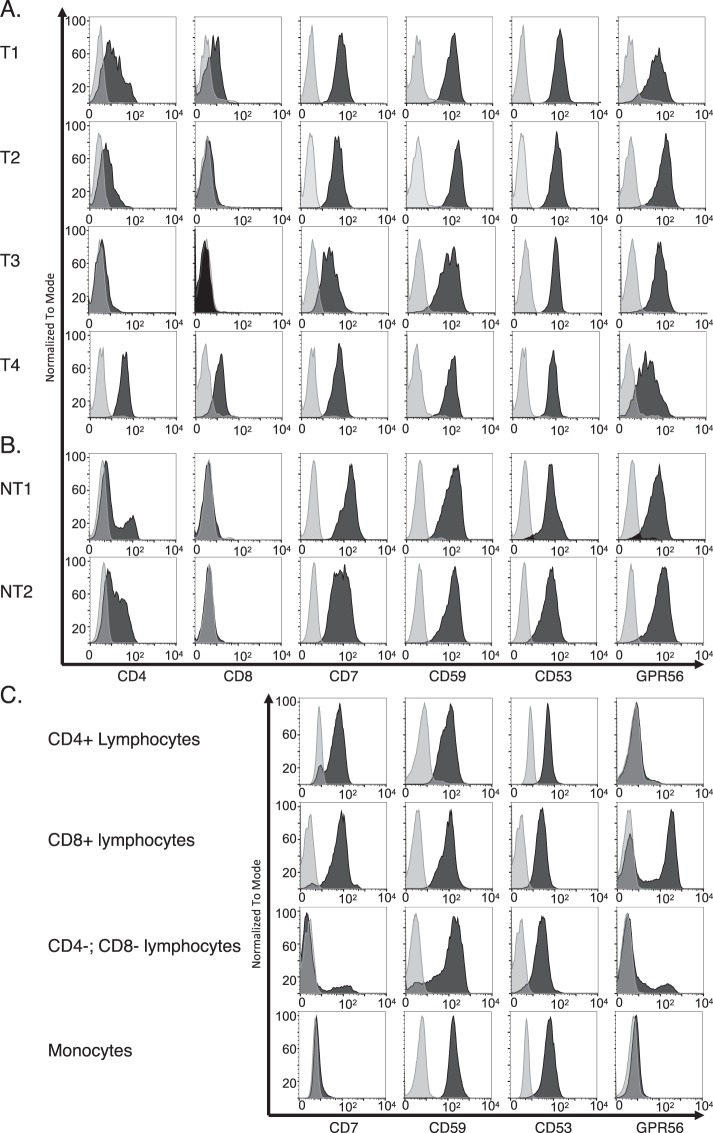
Expression levels of candidate target surface proteins on primary human leukemia samples and normal blood lymphocytes. Flow cytometry analysis of using antibodies detecting either CD7, CD53, CD59a and GPR56 was carried out on four primary human T-ALL harboring the translocation of the *LMO2* gene (T1-T4; see Supplementary Table S6) (panel A) and two human T-ALL lacking *LMO2* translocation (panel B) or different populations of leucocytes from normal peripheral blood mononuclear cells (panel C). Histograms show data for CD45-expressing (common leucocyte antigen) cells: stained cells in black and cells unstained or stained with isotype control in grey. The flow cytometry analysis results for additional and non-translocated human T cell leukemia are shown in Supplementary Fig. S6. In the flow cytometry for the human peripheral blood mononuclear cells (PBMC) (panel C) lymphocytes or monocytes were sorted according to their size and granularity properties. The lymphocytes population was gated according to CD4 expressing cells, CD8 expressing cells or lymphocytes lacking both CD4 and CD8 expression. Monocytes were positive (low) for CD4 expression.

While evaluating the RNA-seq transcriptome data for our human T-ALL samples, we also found a consistent, high expression level of the CD7 marker in RNA-seq (Fig. 2D). CD7 has been highlighted before as a T-ALL target^16^ and the CD7 mRNA in the human T-ALL provides a potential additional marker for the leukemia initiating cells although we cannot evaluate the occurrence of CD7 in the mouse since the mouse gene has eluded characterization in murine T cells^17,18^. However, in each of the TPM comparisons, the human T-ALL samples showed persistent expression, at high levels, of mRNA for the three CD markers that are expressed in the *Lck-Lmo2* mouse DN LICs (GPR56, CD53 and CD59a) and the marker (CD7). These RNA-seq data were verified at the protein level by flow cytometry with the four T-ALL carrying LMO2-translocations (two of which were included in the RNA-seq analysis) and non-translocated samples (T1-T4, NT1-2 and NT3-NT11 respectively, Figs 4A,B and S6). Further, we analyzed published data on 264 samples of pediatric and young adult T-lineage ALL^11^ and show as box plots of the mRNA expression for five markers (together with LMO2) (Supplementary Fig. S7).

We further examined the normal human expression of the four markers using flow cytometry of normal human peripheral blood mononuclear cells (PBMC) (Fig. 4C) to compare tumor with normal cell expression. The CD4+ T cells express CD7, CD53 and CD59a but not GPR56 or CD25 while the CD8+ T cells express all the proteins except CD25. The monocyte population does not show CD7 or GPR56 expression. Of the markers highlighted in this study, GPR56 and CD25 are not expressed on CD4+ cells.

### The LIC antigens can co-localize at the cell surface with CD25

Overall, the expression of these markers (GPR56, CD7, CD53, CD59a) suggests that these are targets for both the overt tumors and the LICs. When we compared the relative mRNA levels of the three markers in DN of transgenic and wild type T cells, we found that the LMO2-expressing transgenic T cells have higher levels at each DN stage (Supplementary Table S8B,C). Further, the levels of the three mRNAs disappear in wild type DN T cells more rapidly in the LMO2-expressing transgenic pre-leukemic DN T cells. Considering the distribution of CD25 and GPR56 in the mouse model and in the human T-ALL, the finding that GPR56 is among the proteins that scores highly in stem cell characteristics on acute leukemia^15,19^, these two markers may be suitable for dual targeting of LICs.

**Figure 5:**
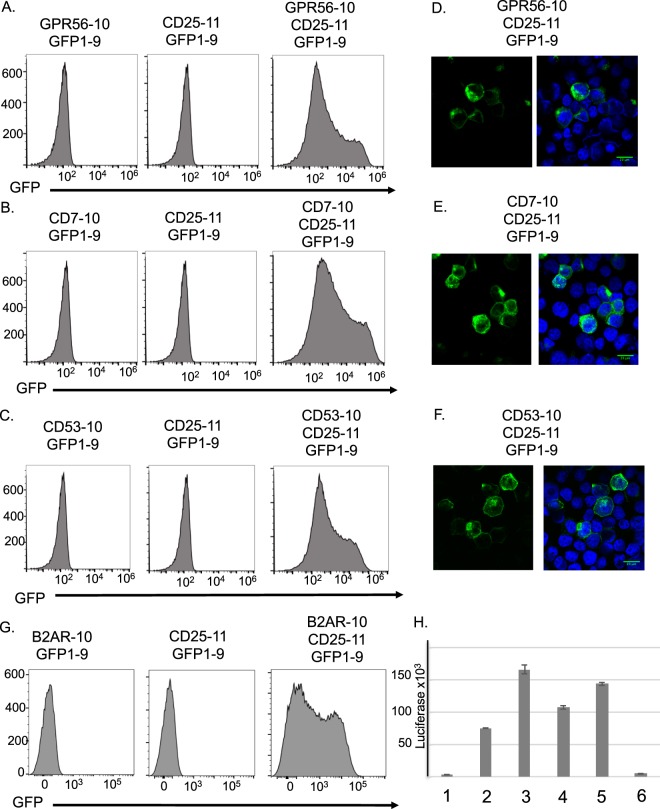
Co-association of surfaceome candidates using split GFP complementation assay. Flow cytometry and confocal microscopy were performed with HEK293T cells co-transfected with antigens fused to GFP10 or GFP11 and GFP1-9. Cells co-transfected with GPR56-(G4S)3-GFP10, CD25-(G4S)3-GFP11 and GFP1-9 (**A**), CD7-(G4S)-3GFP10, CD25-(G4S)3-GFP11 and GFP1-9 (**C**) and CD53-(G4S)3-GFP10, CD25-(G4S)3-GFP11 and GFP1-9 (**E**) were analyzed by flow cytometry. The left-hand and central panels represent negative controls, in which either the GFP10 or GFP11 construct was omitted from the transfection. HEK293T cells co-transfected with GPR56-(G4S)-3-GFP10, CD25-(G4S)3-GFP11 and GFP1-9 (**B**), CD7-(G4S)-3-GFP10, CD25-(G4S)3-GFP11 and GFP1-9 (**D**) and CD7-(G4S)-3-GFP10, CD25-(G4S)3-GFP11 and GFP1-9 (**F**) were stained with DAPI (blue) and the localization of GFP (green) was analyzed by confocal microscopy. A scale bar of 20 µm is included on the merged images. Panel H shows data from an MaMTH assay using HEK293T-5XGAL4UAS-luciferase cells^21,22^. The cells were transfected with plasmids expressing CD25-CUB-TF only (1) or CD25-CUB-TF with either GPR56-NUB (3), CD7-NUB (4), CD53-NUB (5) or B2AR642-NUB (6) and the luciferase activity was measured after 48 hours. A plasmid expressing a GAL4-VP16 fusion protein was used as a positive control (2).

We developed a novel assay to determine if GPR56 and other LIC biomarkers could co-locate on the cell surface with CD25 molecules consistent with capability for binding by bispecific antibodies for internalization. We adapted the protein complementation assay comprising three parts of the green fluorescent protein (GFP) binding to reconstitute GFP and thus fluorescence detectable by flow cytometry or microscopy^20^. Accordingly, we expressed CD25 protein fused to helix 11 of GFP (CD25-11) and either GPR56, CD7 or CD53 fused with helix 10 of GFP (GPR56-10 etc.). Fusion proteins were expressed in HEK293T cells together with the GFP1-9 sub-fragment and GFP signal was determined using flow cytometry (Fig. 5A–C). Each transfected plasmid was separately validated by detection of the relevant protein at the cell surface by flow cytometry (data not shown). GFP was, however, only reconstituted by co-expressing GFP1-9 with CD25-11 together with any one of GPR56-10, CD7-10 or CD53-10. As a control, the beta-2 adrenergic receptor (B2AR) protein was also tested and the reconstitution of GFP was observed (Fig. 5G). The tripartite protein complementation was confirmed at the cell surface shown by confocal microscopy where we observed plasma membrane associated GFP (Fig. 5D–F).

We further confirmed these results using the mammalian-membrane two-hybrid (MaMTH) assay^21,22^. Using this assay, we observed luciferase activation with the CD25-CUB-TF bait co-expressed with GPR56-NUB, CD7-NUB or CD53-NUB, as we had seen with the GFP complementation, but not with B2AR-NUB (Fig. 5H). B2AR-NUB was confirmed at the cell surface of transfected 5xGAL4UAS-luciferase reporter HEK293T cells by flow cytometry (Supplementary Fig. S8). Thus, two independent interaction assays show that CD25 antigen can associate at the cell surface with GPR56, CD7 or CD53. These data suggest that the arrangement of T cell surfaces expressing GPR56, CD7 or CD53 with CD25 will allow the pairs of proteins to co-localize sufficiently to represent authentic dual targets for bsAb.

### Internalization of CD25 after interaction with the GPR56 LIC biomarker

**Figure 6:**
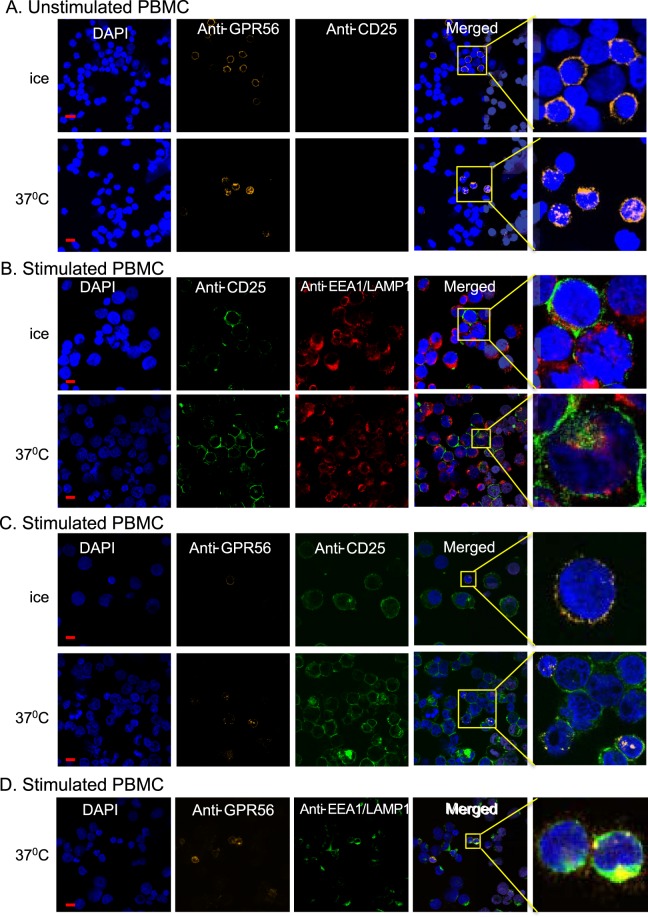
The distribution of the biomarkers CD25 and GPR56 in PBMC after interaction with antibody. PBMCs were stained with DAPI and incubated with anti-CD25 antibody and/or anti-GPR56 antibody. The cells were analyzed by confocal microscopy and images shown at x63 magnification except the right hand panels that were enlarged as indicated. (**A**) PBMC stained with anti-CD25 antibody coupled to Alexa Fluor 488 (green) and anti-GPR56 antibody coupled to PE (orange), and incubated for 2 hours on ice or at 37 °C. (**B**) PHA-stimulated PBMC stained with anti-CD25 antibody coupled to Alexa Fluor 488 (green) and incubated for 2 hours on ice or at 37 °C. The endosomal marker EEA1 and the lysosomal marker LAMP1 were detected by incubating with rabbit antibody and stained using Alexa 594-labeled (red) secondary antibody. (**C**) PHA-stimulated PBMC stained with anti-CD25 antibody coupled to Alexa Fluor 488 (green) and anti-GPR56 antibody coupled to PE (orange), and incubated for 2 hours on ice or at 37 °C. (**D**) PHA-stimulated PBMC stained with anti-GPR56 antibody coupled to PE (orange) and incubated for 2 hours at 37 °C. The endosomal marker EEA1 and the lysosomal marker LAMP1 detected by incubating with rabbit antibody and stained using Alexa 488-labeled (green) secondary antibody. The red scale bar of 10 μm is shown on the DAPI stained images.

The use of antibody-based therapies, such as ADC, aimed at the LICs or bulk tumor cells, requires the endogenously expressed antigens to move on the membrane and undergo endocytosis when bound by antibody. This membrane movement capability was analyzed in normal human (PBMC, either un-stimulated or stimulated with phytohaemagglutinin (PHA)). We could detect surface GPR56 but not CD25 protein on the normal, unstimulated PBMCs (Fig. 6A) and incubation with the corresponding antibody at 37 °C resulted in internalization of this GPR56. PHA-stimulated PBMC express both CD25 and GPR56 (Fig. 6B–D, note the different cell size in un-stimulated and stimulated cultures). Unlike the internalization of GPR56 on the majority of cells, few cells show internal fluorescence from anti-CD25 antibody after incubation at 37 °C (Fig. 6B). However, in these cells, the CD25 signal did not co-localize with signals from antibodies binding the early endosomal marker EEA1 or the lysosomal marker LAMP1 (see the enlarged images Fig. 6B). The failure of CD25-immune complexes to internalize was investigated with mouse thymocytes. We observed CD25 internalization in mouse thymocytes but, similar to human cells, the fluorescent signal in the cells did not appear to co-localize with anti-EEA1 or anti-LAMP1 antibody signals (Supplementary Fig. S9).

As the human CD25 antigen does not undergo antibody-mediated endocytosis, we investigated whether the internalization of GPR56 could carry along CD25. Thus, we implemented the tripartite GFP complementation method^20^ to assess whether CD25 could move inside cells when co-associated with GPR56. We observed GFP signals as expected for the antigens on the cell surface but also inside the cells (see Fig. 5). This later signal was observed although the transfected plasmids coded for the membrane-associated proteins including the leader peptide. Hence, CD25 can apparently re-internalize after surface expression (and removal of the leader peptide) when appropriately cross-linked with other antigens (Supplementary Fig. S10).

**Figure 7:**
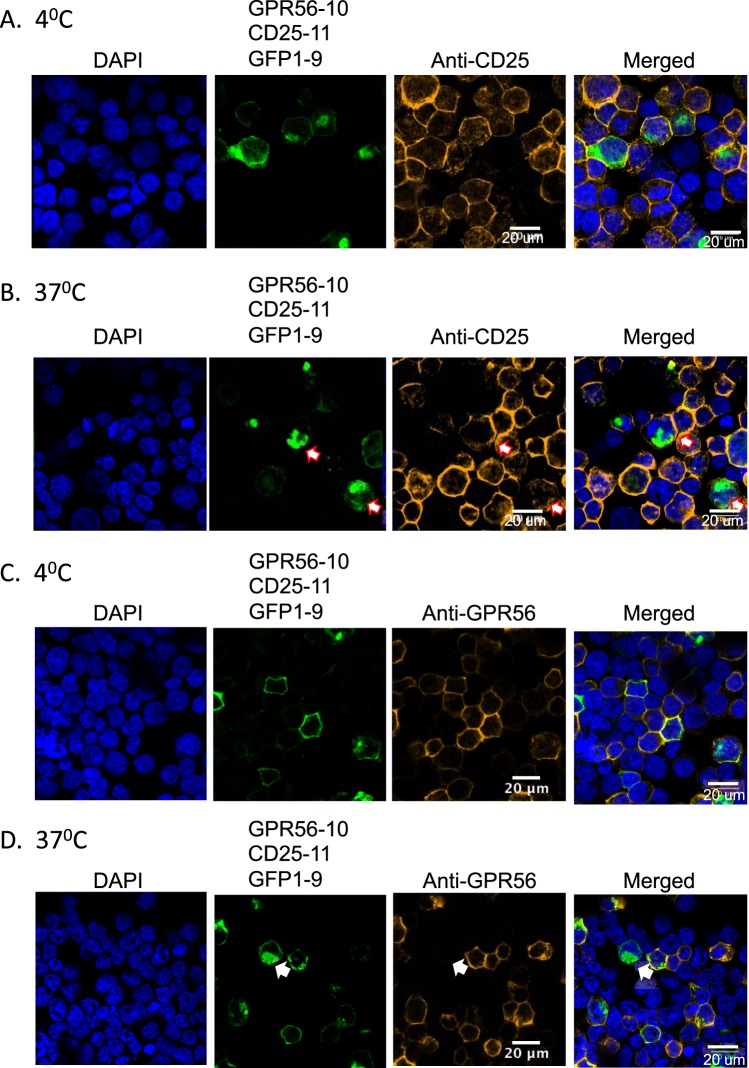
Co-localization of CD25 and GPR56 using GFP-complementation in HEK293T cells. HEK293T cells were co-transfected with GPR56-(G4S)3-GFP10, CD25-(G4S)3-GFP11 and GFP1-9, incubated with PE-anti-human CD25 or PE-anti-human GPR56 and stained with DAPI (blue). The localization of GFP (green) was analyzed by confocal microscopy using a x63 objective. The co-transfected cells were incubated with PE-anti-human CD25 (orange) at 4 °C (**A**) and 37 °C (**B**) and with PE-anti-human GPR56 (orange) at 4 °C (**C**) and 37 °C (**D**). A scale bar of 20 μm is included on the PE and merged images. The white arrows depict cells where surface antigen levels decrease as a result of GFP internalization.

Confocal microscopy revealed surface associated GFP (Fig. 7A,B) and the anti-CD25 is clearly co-localized to the cell surface, whether or not the cells were incubated at physiological temperature. We noted however many cells that had been incubated at 37 °C and had GFP fluorescent signal inside the cells also lacked cell surface staining when tested with anti-CD25 antibody, suggesting that the cross-linked complex (comprising GPR56-10/CD25-11/GFP1-9) can move from the membrane into cells by endocytosis also carrying the anti-CD25 antibody (Fig. 7B, indicated by white arrows).

Further, we could detect distinct GFP “caps” together with some internal GFP signal on the cells expressing GPR56-10, CD25-11 and GFP1-9 (Fig. 7A,C) indicative of the accumulation of GFP deposits (internalization) of the tripartite complex without antibody. When the same transfected cell populations were incubated with anti-GPR56 antibody, however, we could again observe cells where the GFP signal occurs inside the cells but with little remaining anti-GPR56 staining on the cell surface (Fig. 7D, indicated by the white arrows). These data support the hypothesis that GPR56 internalizes into cells by endocytosis and in doing so, can carry CD25 protein that is associated with it.

## Discussion

### Surfaceome description using RNA-seq data analysis of low cell numbers

The importance of describing the protein molecules on the surface of specific cell types is pivotal for all biological phenomena because cell-cell and cell-ligand interactions are critical in normal development, homoeostasis and disease development. A direct approach for analyzing the surfaceome is by proteomic methods but obtaining the complete proteome of small numbers of cells remains a major challenge^4^ and requires specialist equipment. As a surrogate, and alternative to proteomic approaches, we developed a simple bioinformatics approach to the surfaceome based on RNA-seq deep sequencing data using our database of cell surface and secreted proteins^1^. The RNA-seq approach allows sufficient information to be generated, even from limited sample sizes. RNA-seq only measures the transcriptome level and the correlation between RNA and protein may be low for a number of reasons, such as stability and lifetime difference of the two types of molecules and post-transcriptional/post-translational modifications. Therefore, the validation of the targets predicted from RNA-seq results is necessary by subsequent protein analysis. In our experimental approach, we have used flow cytometry with specific antibodies to exemplify and validate the RNA-seq profiles, as well as features of bi-molecular interaction at the cell surface that may be important to implement antibody-mediated therapy strategies.

A feature of the RNA-seq surfaceome database approach is that it allows comparative analysis of low cell numbers. Thus in our analysis we have compared purified CD4; CD8 negative thymocytes from wild type (healthy) mice with those from a leukemia pre-disposition model and corroborated with a panel of human T-ALL samples. Analogous comparisons between tumour and normal cell equivalents in other human leukemias and in carcinomas, or in other diseases including neuropathies, will be important to identify surface markers for diagnosis, prognosis and therapy. With the expansion of new strategies, such as CAR-T and antibody-drug conjugates (ADCs), the cancer cell surfaceome will become increasingly important to discover new surface target molecules. An additional distinctive aspect of using the RNA-seq surfaceome database approach is that it can be applied to meta-analysis of published RNA-seq data that may not be accompanied by proteomics. A final advantageous aspect of the RNA-seq approach is that it will be applicable to the burgeoning amount of single cell RNA-seq data that will be published and for which proteome data will not be available.

### Surfaceome RNA-seq analysis of LMO2-dependent T cell neoplasia

T cell acute lymphoblastic leukemia (T-ALL) is a malignancy that occurs in all age groups, with higher incidence in early age and increasing frequency after about 70 years of age^23^. The development of T cell acute lymphoblastic leukemia late in life is difficult to treat since targeting T cell populations is associated with immuno-suppression since there is limited replenishment of functional T cells^24^. Infants and children can be successfully treated with high dose chemotherapy but with adverse side effects^25^, implying that chemo-resistant cancer initiating cells (minimal residual disease) persist after primary treatment and propagate relapsed disease. Effective targeting of chemo-resistant cells with developing immuno- therapies requires knowledge of the cell surface.

As the asymptomatic phase is inaccessible in the human T-ALL, there has been reliance on animal models to dissect these crucial early stages. While it has been possible to establish LSC in human acute myeloid leukemia transplantation models^26^, it has been difficult to maintain T-ALL biopsies in culture, and derive cells for limiting dilution to identify the LSC^27^. We have used a mouse model of human T-ALL driven by the T cell oncogene *LMO2*^6,8,28,29^, characterized by a differentiation block in the T cell progenitors at the DN stage^6,7,29^ to assess the surfaceome using the RNA-seq method because there are limited cell numbers of the subclasses of thymocytes and there is no human equivalent T-ALL LIC/LSC available.

In our study, next generation RNA-seq^30^ was carried out with small numbers of purified subsets of DN thymocytes from age-matched LMO2 transgenic or wild type mice, showing many mRNAs elevated in the transgenic *Lck-Lmo2* DN2/3 cells compared to normal DN cells (Fig. 1). Our data show the co-association of the markers GPR56, CD25, CD53 and CD59a on the LICs in this model and that these markers persist in the overt T-ALL. GPR56 seems particularly important as it has recently been included in a group of 17 proteins with prognostic significance in acute myeloid leukemia (AML) and expressed on AML LSCs^15^. GRP56 is the only one of the 17 proteins that coincides between the AML LSC study and our T-ALL study. This is partly because the Ng *et al*.^15^ study involved proteins that secreted but also included intracellular proteins such as transcription factors while our study was focused on the surfaceome. Only GPR56 appears in both studies but this may not necessarily be a significant difference because of the small numbers of relevant mRNAs and also the AML stemness group may reflect protein involved more in the myeloid lineage leukemia stemness than in the lymphoid lineage.

**Figure 8:**
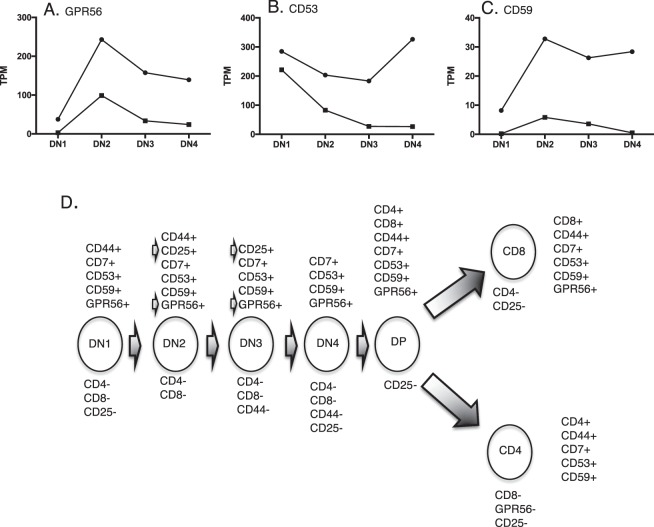
Range of CD-marker expression in thymocyte populations and LSC represented by *Lck-Lmo2* thymus cells. TPM values of each mRNA were computed from the DN cells of asymptomatic, pre-leukaemic *Lck-Lmo2* or age-matched wild type mice for GPR56 (**A**), CD53 (**B**) and CD59 (**C**). Closed circles are *Lck-Lmo2* values, closed squares are wild type values. Panel D summarizes the expression patterns of the surface proteins analyzed in this study, during T cell development from immature DN1 cells through to single positive CD4 or CD8, T cell receptor expressing T cells. Two key markers, CD25 and GPR56, first expressed on the LICs are arrowed where their co-expression is found and that could be employed to target the LMO2-dependent T-ALL LSC.

The co-expression and co-association of GPR56 and CD25 on the LICs suggest these as potent pair of markers for antibody-based and immuno-therapy in T-ALL management. While the occurrence of GPR56, CD53 and CD59a on leukemia cells is known^31–36^, GPR56, CD53 and CD59a are highly expressed in the thymocytes of *Lck-Lmo2* mice relative to wild type thymocytes (Fig. 8A–C) and are also on the overt human tumors in addition to CD7. Figure 8D summarizes the expression of these markers in T cell differentiation suggesting interesting options for cancer therapy by targeting both LICs and bulk cancer cells.

### Co-localization and co-internalization of surface antigens

CD25 alone has the advantage as a T-ALL LIC target because of its absence except activated T cells and a sub-population of memory CD8+ cells and there is little evidence of expression outside the immune system http://www.proteinatlas.org/ENSG00000134460-IL2RA/tissue. However, antibody-mediated therapy, such as ADC, requires internalization of the immune complex of the ADC-antigen. CD25 and GPR56 are co-expressed on the mouse LMO2 LIC model and dual targeting of GPR56 and CD25 is an intriguing possible approach because we observed that the co-association of GPR56 with CD25 triggers CD25 entry into cells along with the GPR56. A bispecific anti-GPR56-CD25 ADC is therefore an intriguing possibility. Multiple-specific markers have led to further developments in targeting leukemia using bi-specific^37,38^ or tri-specific agents^38,39^. The T-ALL LIC surfaceome is an important resource for antibody-mediated regimes such as these. With the incorporation on new technologies (bsAbs, biTEs, CAR-T), use of anti-CD25 in bispecific format with anti-GPR56 could overcome both the poor internalization of CD25 and off-target toxicity problems. The targeting of LICs with anti-CD25-GPR56 bispecific immuno-nanoparticles is an exciting future option for instance for delivering high affinity, high specificity intracellular antibody fragments (macrodrugs^40^). Developments in nanotechnology are making inroads into the problem of macromolecule delivery into cells^41^ and we have recently developed nanoparticles that can deliver mRNA to cells^42^. As progress in these entities improve, immuno-nanoparticles should increase in importance in leukemia management.

## Methods

### Thymocyte purification and RNA-seq deep sequencing

Thymus biopsies from *Lck-Lmo2* and wild type mice at P90 were crushed to obtain single cell suspensions. DN1, DN2, DN3 and DN4 cells were sorted on the basis of surface markers: CD90+ CD4− CD8− CD25− CD44+ (DN1) CD90+ CD4− CD8− CD25+ CD44+ (DN2), CD90+ CD4− CD8− CD25+ CD44− (DN3), and CD90+ CD4− CD8− CD25− CD44− (DN4). RNA was extracted and quality was assessed with an Agilent RNA 6000 Pico Assay on Agilent 2100 Bioanalyzer. Sequencing library generation and sequencing were performed by the High-Throughput Genomics Group at the Oxford Wellcome Trust Centre for Human Genetics. Deep sequencing data were analyzed as detailed in Methods Online.

### RNA-seq data surfaceome analysis

Deep sequencing data were trimmed using Trim Galore! to remove unwanted adapter sequences http://www.bioinformatics.babraham.ac.uk/projects/trim_galore/. mRNA expression levels were estimated using Kallisto^43^, which uses a pseudo-alignment algorithm to probabilistically assign individual sequences to mRNAs. For this purpose, we obtained the mouse mRNA collection from Ensembl (version 82)^44^. The Kallisto alignment was performed using the default settings, giving one estimate per mRNA and per sample. These estimates were subsequently analyzed using the statistical software R https://www.r-project.org/. Extraction of expressed mRNAs encoding for known and presumptive cell surface proteins was carried out as previously described^1^ using our surfaceome database http://www.imm.ox.ac.uk/surfaceome-database. Correlation analysis and Principal Component Analysis were carried out in R. The expression classes In Figure 1tiles were defined by TPM as: 0 – less than 0.1 TPM; LOW – between 0.1 and 1 TPM; MED – between 1 and 10 TPM; HIGH – more than 10 TPM. Each tile was produced by the number of mRNAs (with grey corresponding to lower values and red to higher value).

### Surface protein analysis of mouse T cells by flow cytometry

Single cell thymus cell suspensions were prepared and red cells lysed. 3-5 × 10^6^ thymocytes are re-suspended in PBS, 1% FBS and stained with indicated antibodies. Flow cytometry was performed using CyAn™ ADP Analyzers. The antibodies were from BD Biosciences unless otherwise stated (PE-Cy™7 Rat Anti-Mouse CD4: 561099, FITC Rat Anti-Mouse CD8a: 553030, V450 Rat Anti-Mouse CD90.2: 561643, APC Rat Anti-Mouse CD25: 561048, V500 Rat Anti-Mouse CD44: 560781, PE Anti-Mouse CD59a: 143104 from Biolegend, BB515 Rat Anti-Mouse CD53: 565288).

### Surface expression of surface markers on primary human T cell leukemia

Human primary T-ALL samples were obtained from Birmingham Children’s Hospital with informed and written consent obtained from the patient or parent or legal guardian as appropriate, in accordance with the UK Regulations under the NHS Research Governance Framework and with the Trent Research Ethics Committee approvals of the Childhood Cancer and Leukaemia Group (CCLG) Tissue Bank (MREC 08/H0405/22 and 08/H1208/40). Primografts were generated by passage through NSG immunodeficient mice (NOD.Cg-*Prkdc*^*scid*^ *Il2rg*^*tm1Wjl*^/SzJ) in accordance with UK Home Office guidelines, Schedule 1, project license number PPL 30/2094. Mononuclear cells from primary human T-ALL mononuclear cells were isolated from bone marrow or peripheral blood samples by gradient centrifugation. 10^6^ cells were intravenously injected via the tail vein into NSG immunodeficient mice ((NOD.Cg-*Prkdc*^*scid*^ *Il2rg*^*tm1Wjl*^/SzJ) and engrafted cells obtained from enlarged spleens (after approximately 4–6 weeks) and frozen in viable state. Cytogenetic analyses were performed by the West Midlands Regional Genetics Laboratory. For flow cytometry, T-ALL primograft cells were thawed and washed in PBS, 1% FBS. 1 × 10^6^ cells was stained in PBS, 1% FBS with antibodies detecting CD45 (lymphocyte common antigen) and those detecting the markers CD4 (FITC), CD8 (PE), CD7 (FITC), CD59a (PE), CD53 (PE), GPR56 (PE) and isotype controls. Anti-human antibodies for flow analysis were purchased from BD Pharmingen (see below) with the exception of anti-human GPR56 and isotype control (PE Mouse IgG1, κ isotype control) from Biolegend.

Cells were analyzed by flow cytometry with a CyAn™ ADP Analyzer (Beckman Coulter).

Mean fluorescent values were calculated using the FlowJo software gated in the stained sample and in the control (sample stained for CD45 and isotype for PE). Samples with non-homogeneous populations were excluded from this analysis. P values between translocated and non-translocated TALL were calculated with the Mann Whitney test (non-parametric t test).

Antibodies catalogue numbers from BD biosciences: FITC mouse anti human CD59: 560954; APC mouse anti human CD8: 561952; V450 mouse anti human CD45: 560367; PE mouse anti human CD53: 555508; PE mouse anti human CD8: 555635; FITC mouse anti human CD4: 555346; PE mouse anti human CD4: 555347. The Biolegend antibodies used: anti-GPR56 antibody (Biolegend 358203); anti-CD25 antibody (Biolegend 302615).

The gene expression values (FPKM) of LMO2, GPR56, CD53, CD59, CD7 and IL2RA in 264 paediatric T-ALL samples were retrieved from the supplementary information (Table S5 RNA-seq FPKM) available from the online version of paper of Liu Y *et al*.^11^. A boxplot was created to visualise the distribution of the expressions values for each gene using R.

### Analysis of PBMC surface and antigen internalization

A frozen vial of human peripheral blood mononuclear cells (PBMC) from a healthy donor was thawed and washed in PBS 1%FBS. 10^6^ cells were stained with the following combination of antibodies: CD8 APC, CD4 PE and CD7 FITC or CD59 FITC; the control sample was stained with CD8 APC and CD4 PE. CD8 APC, CD4 FITC and CD53 PE, GPR56 PE, or PE Isotype control (control sample).

PBMC from normal donors were cultured in RPMI1640 + 10% FBS for 24 hours and harvested by centrifugation (300 × g, 7 minutes). Cell viability and number were determined with a haemacytometer after staining with trypan blue. The cells were re-suspended in PBS + 10% FBS at 10^7^ cells/ml. An aliquot of 2 × 10^6^ cells was incubated with 5 μl of Alexa Fluor 488 coupled anti-CD25 antibody (Biolegend 302615) and/or 5 μl of PE coupled anti-GPR56 antibody (Biolegend 358203) on ice for 30 minutes. The cell suspension was split into two aliquots of 10^6^ cells and one aliquot was kept on ice while the other was incubated at 37 °C for 90 minutes. Cells from both aliquots were washed once with 5 ml ice-cold PBS + 10% FBS and collected by centrifugation at 500 rpm for 5 minutes at 4 °C (Thermo Scientific Cytospin 4 Cytocentrifuge). Cells were fixed with 4% formaldehyde at room temperature for 15 minutes and transferred onto coverslips by cytospin (Thermo Scientific Cytospin 4 Cytocentrifuge). Permeabilization was carried out in 0.5% Tween 20 in PBS for 15 minutes at room temperature followed with 30 minutes incubation in PBS with 10% FBS and 0.1% Tween blocking buffer.

For PBMC incubated with single antibody, either anti-CD25 or anti-GPR56, the endosomal marker EEA1 or lysosomal marker LAMP1 were detected by incubating cells on coverslips with anti-EEA1 (Cell Signaling 3288 S) and anti-LAMP1 (Cell Signaling 9091 S) antibodies at 1:150 dilution for 1 hour, followed by incubation with Alexa Fluor 594 or Alexa Fluor 488 coupled anti-rabbit secondary antibody (Invitrogen A-21207; Invitrogen A-11034) at 1:200 dilution for 1 hour. The coverslips were washed three times with PBS + 10% FBS and mounted using mounting medium containing DAPI (SouthernBiotech 0100-20). Slides were analyzed using a Zeiss 880 Inverted Confocal Microscope with a x63 objective.

### PHA stimulation of PBMC

PBMC from normal donors were cultured in RPMI1640 + 10% FBS for 5 hours in a 75 cm^2^ cell culture flasks. Phytohaemagglutinin (PHA, SIGMA-ALDRICH L9017-1MG, prepared at 1 mg/ml with PBS) was added to the PBMC culture to the final concentration of 10 μg/ml. Cells were stimulated by the PHA at 37 °C, 5% CO_2_ for 72 hours.

### Analysis of antigen internalization by mouse thymocytes

Thymus of an *Lck-Lmo2* transgenic mouse was removed and dispersed into single cell suspension by filtering through a 40 μm cell strainer (Falcon 352340). Cells were harvested by centrifugation (400 × g, 5 minutes), re-suspended in 1 ml of red cell lysis buffer (17 mM Tris-HCl, 144 mM NH_4_Cl, pH 7.5) and incubated at room temperature for 15 minutes. The lysis was stopped by adding 20 ml of PBS + 10% FBS. Cells were pelleted again by centrifugation (400 × g, 5 minutes) and re-suspended with PBS + 10% FBS at a cell concentration 10^7^ cells/ml. An aliquot of 2 × 10^6^ cells was incubated with 5 μl of APC coupled anti-CD25 antibody (BD 561048) on ice for 30 minutes. The cell suspension was split into two aliquots of 10^6^ cells and one aliquot was kept on ice while the other was incubated at 37 °C for 2 hours. Cells from both aliquots were washed once with 5 ml ice-cold PBS + 10% FBS and collected by centrifugation (400 × g, 5 minutes) at 4 °C. Cells were fixed with 4% formaldehyde at room temperature for 15 minutes and transferred onto coverslips by centrifugation at 500 rpm for 5 minutes (Thermo Scientific Cytospin 4 Cytocentrifuge). Permeabilization was carried out in 0.5% Tween 20 in PBS for 15 minutes at room temperature followed with 30 minutes of incubation in PBS with 10% FBS and 0.1% Tween blocking buffer.

For co-location of internalized antibody with the endosomal marker EEA1 or the lysosomal marker LAMP1, mouse thymus T cells that had been incubated with antibodies to detect the relevant surface markers were detected by incubating cells on coverslips with anti-EEA1 (Abcam ab2900) and anti-LAMP1 (Abcam ab24170) antibodies at 1:200 dilution for 1 hour, followed by incubation with Alexa Fluor 488 coupled anti-rabbit secondary antibody (Invitrogen A-11034) at 1:200 dilution for 1 hour. The coverslips were washed three times with PBS + 10% FBS and mounted using mounting medium containing DAPI (SouthernBiotech 0100-20). Slides were analyzed using a Zeiss 880 Inverted Confocal Microscope with a x63 objective.

### CS-GFP complementation analysis

Expression plasmid preparation: Total RNA was extracted from Jurkat cells with TRIzol® reagent (Invitrogen), according to the manufacturer’s protocol. cDNA was synthesized using SuperScript™ III Reverse Transcriptase (Invitrogen) with Oligo d(T)_18_ mRNA primers (New England BioLabs), following the manufacturer’s instructions. GPR56, CD7, CD25 and CD53 were PCR amplified from the cDNA and were subsequently sub-cloned into the pBlueScriptII SK+ vector (Agilent). Oligonucleotides encoding the GGGGSGGGGSGGGGS ((G4S)3) linkers were synthesized (Sigma), annealed and sub-cloned into the antigen containing pBlueScriptII SK+ vectors. DNA encoding GFP10 and GFP11 was synthesized (Eurofins Genomics) and added to the constructs to give GPR56-(G4S)3-GFP10, CD7-(G4S)3-GFP10, CD53-(G4S)3-GFP10 and CD25-(G4S)3-GFP11. GFP1-9 synthesized DNA (Eurofins Genomics) and the antigen-linker-GFP10/11 constructs were PCR amplified and cloned into pEF/myc/cyto (Invitrogen) using PmlI and SalI for mammalian expression. Sequences of the constructs prepared are given in Supplementary Fig. S10.

Transient transfection was according to^20^. HEK293T or DLD-1 cells were maintained in Dulbecco’s Modified Eagle Medium (DMEM) supplemented with 10% FBS, 100 U/mL penicillin and 100 μg/mL streptomycin. Cells were co-transfected with plasmid DNA encoding GFP1-9, CD25-(G4S)3-GFP11 and GPR56-(G4S)3-GFP10, CD7-(G4S)3-GFP10 or CD53-(G4S)3-GFP10 using Lipofectamine 2000 (Invitrogen), according to the manufacturer’s protocol. After 48 hours cells were trypsinized and analyzed for GFP fluorescence using flow cytometry and confocal microscopy. Flow cytometry measurements were performed using an Attune NxT Flow Cytometer (Thermo Fisher Scientific). The data was processed using FlowJo software. For imaging 5 × 10^6^ cells were fixed with 4% formaldehyde for 15 minutes at room temperature before being transferred to a coverslip using a Cytospin Cytocentrifuge (Thermo Fisher Scientific). Mounting media containing DAPI (SouthernBiotech) was pipetted onto the cells and the coverslip was inverted onto a glass slide. Slides were analyzed using a Zeiss LSM 880 Inverted Confocal Microscope (Zeiss) equipped with a x63 oil immersion objective. Data was analyzed using FIJI (Image J).

Antigen internalization analysis: HEK293T cells transfected with pEF-GPR56-(G4S)3-GFP10, pEF-CD25-(G4S)3-GFP11 and pEF-GFP1-9 were trypsinized and re-suspended in PBS + 1% FBS at 1 × 10^7^ cells/mL. Cells were incubated with either 4 μL of PE anti-human GPR56 (Biolegend) or 4 μL of PE anti-human CD25 (Biolegend) on ice for 30 minutes. Half of the cells were transferred to a fresh tube and incubated at 37 °C for 90 minutes, while the remaining cells were retained on ice. Cells were washed with ice-cold PBS + 1% FBS before being fixed with 4% formaldehyde for 15 minutes at room temperature. Cells were transferred to a coverslip using a Cytospin Cytocentrifuge (Thermo Fisher Scientific). Mounting media containing DAPI (Southern Biotech) was pipetted onto the cells and the coverslip was inverted onto a glass slide. Slides were analyzed using a Zeiss LSM 880 Inverted Confocal Microscope (Zeiss) equipped with a 63x oil immersion objective. Data was analyzed using FIJI (Image J).

### Antigen internalization and co-localization

PBMC from normal donors or thymocytes from transgenic mice were incubated with fluorescently labelled anti-CD25 antibody or anti-GPR56 antibody either on ice or at 37 °C for 1 - 2 hours, followed by cytospin, fixation and permeabilization for analysis by confocal microscopy. GPR56, CD7, CD25 and CD53 cDNAs were amplified from Jurkat cells and GFP10 and GFP11 was added to the constructs to give GPR56-(G4S)3-GFP10, CD7-(G4S)3-GFP10, CD53-(G4S)3-GFP10 and CD25-(G4S)3-GFP11. HEK293T or DLD-1 cells were co-transfected with plasmid DNA encoding GFP1-9, CD25-(G4S)3-GFP11 and GPR56-(G4S)3-GFP10, CD7-(G4S)3-GFP10 or CD53-(G4S)3-GFP10 and analyzed for GFP fluorescence by flow cytometry and confocal microscopy. The transfected HEK293T cells were also incubated with PE labelled anti-human GPR56 or anti-human CD25 either on ice or at 37 °C for 90 minutes followed by cytospin and fixation for confocal microscopy.

### Mammalian Membrane Two hybrid (MaMTH) analysis

MaMTH analysis was carried out as described^21,22^. HEK293T cells stably carrying a reporter gene with five *GAL4* upstream activating sequence repeats followed by the luciferase gene (GAL4-UAS-luciferase) were used as a reporter line. The cells were co-transfected with plasmids expressing the CD25-CUB-TF bait together with plasmids expressing prey proteins GPR56, CD7, CD53 and B2A2 fused to the NUB sequence. The cDNA sequence of B2A2 used in the bait construct is shown in Supplementary Fig. S10. After 48 hours, cells were washed with PBS, lysed with 1x Reporter Lysis Buffer (Promega) and frozen at −80 °C for 1 hour. The lysates were analyzed for firefly luciferase using the E1500 Luciferase Assay System (Promega). Cell surface expression of the B2A2-NUB protein was confirmed using flow cytometry with the PE-coupled antibody B2AR (R11E1) (Santa Cruz).

## Supplementary information

Supplementary information
