# Supplementary material for: Surfaceome interrogation using an RNA-seq approach highlights leukemia initiating cell biomarkers in an LMO2 T cell transgenic model
Source: Sci Rep. 2019 Apr 8;9:5760. doi: 10.1038/s41598-019-42214-w (PMC6453905; doi:10.1038/s41598-019-42214-w)
Supplement: Supplementary file 1 — Supplementary information [file 41598_2019_42214_MOESM1_ESM.pdf]

Surfaceome interrogation using an RNA-seq approach highlights leukemia initiating cell biomarkers in an *LMO2* T cell transgenic model

Helio Pais, Katia Ruggero, Jing Zhang, Osama Al-Assar, Nicolas Bery, Ravneet Bhuller, Victoria Weston, Pamela R. Kearns, Cristina Mecucci, Ami Miller & Terence H. Rabbitts*

Supplementary figures 1-10
Supplementary Tables 1-8

Supplementary Figure S1

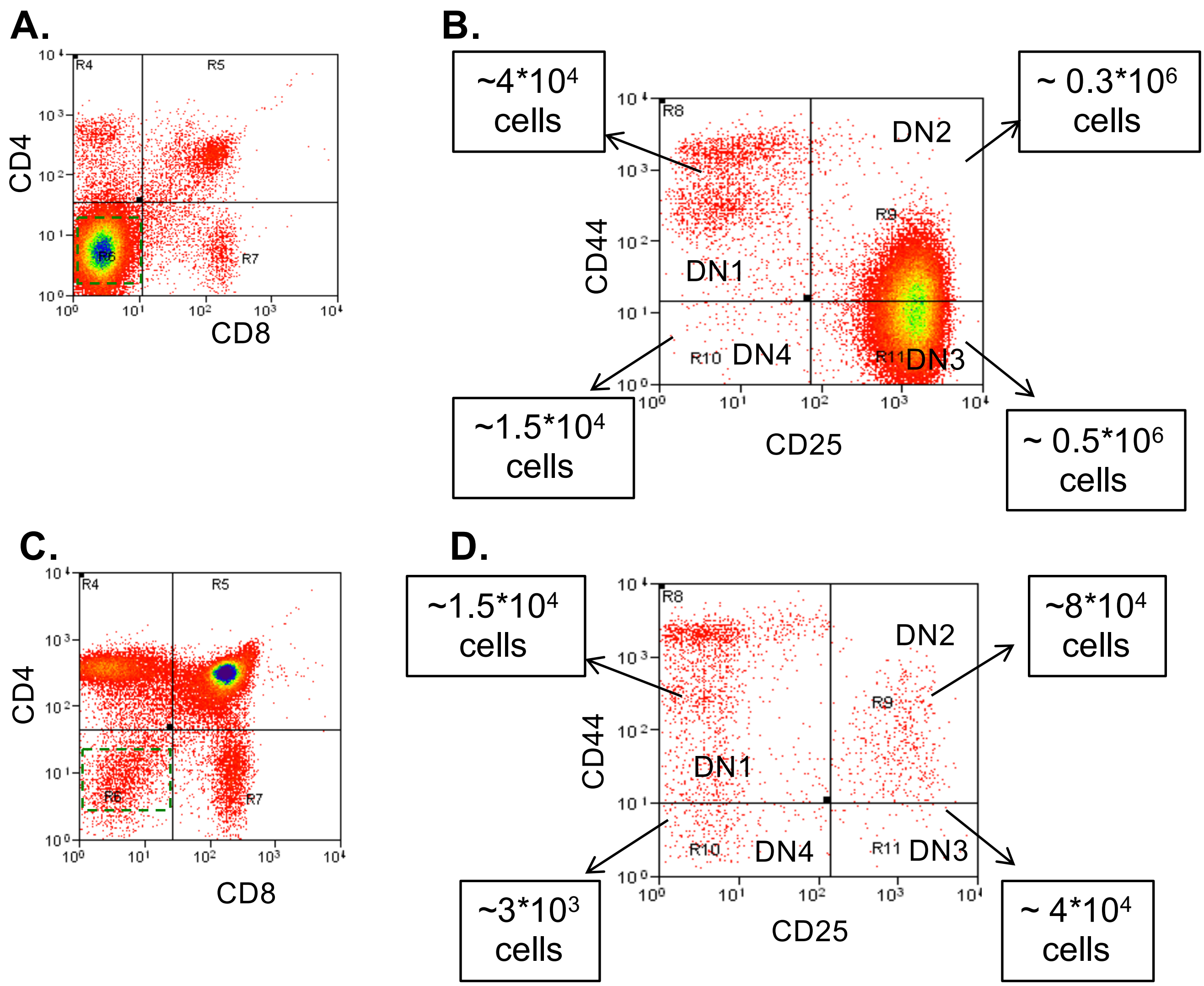

Supplementary Figure S1. Separation of DN thymocytes populations from *Lck-Lmo2* transgenic and wild type thymocytes for RNA-seq

19-21 week old mice (three for each genotype) were used to source thymocytes that separated into the four CD4-CD8- DN populations using CD90, CD4, CD8, CD44 and CD25 antibodies. The DN cells were separated by flow cytometry and RNA made directly from the indicated yields of cells.

A, C, Profile of CD4; CD8 cells from *Lck-Lmo2* and wild type mice respectively

B, D. CD44; CD25 profile and the yield of cells in each sub-type from *Lck-Lmo2* and wild type mice respectively

Supplementary Figure S2

WT, DN1	WT, DN2	WT, DN3	WT, DN4	LckLmo2, DN1	LckLmo2, DN2	LckLmo2, DN3	LckLmo2, DN4	
1.00	0.90	0.89	0.90	0.93	0.89	0.89	0.88	**WT, DN1**
	1.00	0.94	0.93	0.90	0.94	0.93	0.92	**WT, DN2**
		1.00	0.93	0.89	0.92	0.93	0.92	**WT, DN3**
			1.00	0.89	0.93	0.93	0.93	**WT, DN4**
				1.00	0.91	0.90	0.89	**LckLmo2, DN1**
					1.00	0.96	0.94	**LckLmo2, DN2**
						1.00	0.94	**LckLmo2, DN3**
							1.00	**LckLmo2, DN4**

Supplementary Figure S2. Correlation table of RNA populations from DN *Lck-Lmo2* transgenic and wild type thymocytes

The yellow cells show the correlation values between the RNA sub-population isolated from either wild type (for instance, wild type, DN1 vs wild type, DN2) or from *Lck-Lmo2* transgenic, and the blue cells show the correlation between sub-populations of wild type compared to *Lck-Lmo2* transgenic. The dark blue cells show the correlation between corresponding pairs of sub-populations in wild type and *Lck-Lmo2*. The correlation values are high, confirming the high similarity between the expression profiles in the two.

Supplementary Figure S3

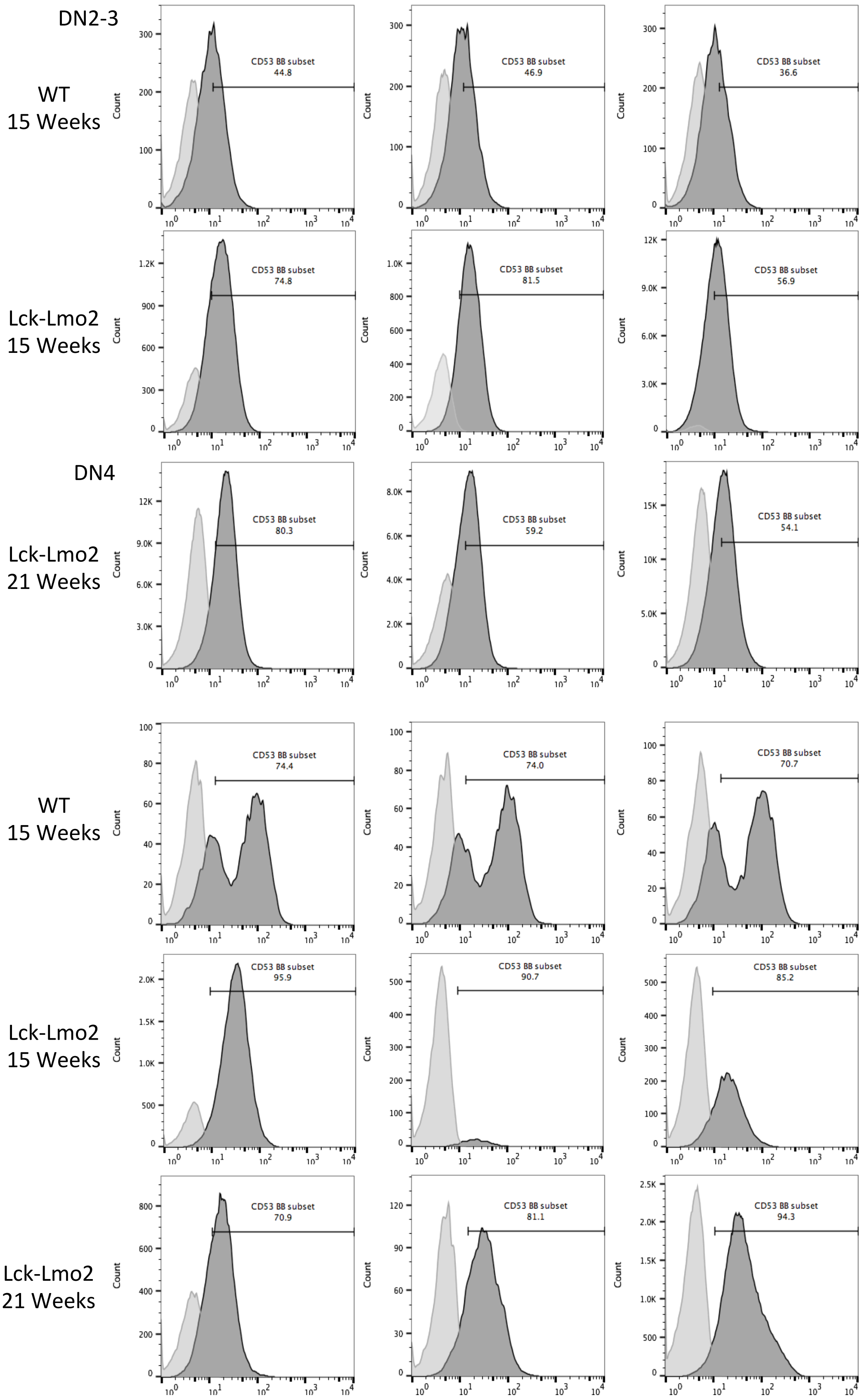

Supplementary Figure S3. Flow cytometry of CD53 expression on asymptomatic *Lck-Lmo2* mouse DN thymocytes.

Thymocytes were prepared as single cell suspensions from *Lck-Lmo2* mice or wild type equivalents and analyzed by flow cytometry with anti-CD53 antibodies. Expression of CD53 was measured in three wild type or three asymptomatic *Lck-Lmo2* mice (wild type mice, 15 weeks old; Lck-LMO2, 3 mice 15 weeks old, 3 mice 21 weeks old). The plots show the results for CD53-expressing cells (dark grey) compared with the isotype control (light grey) for pooled DN2-3 and for DN4 cells. The x-axis shows fluorescence on a log scale.

Supplementary Figure S4

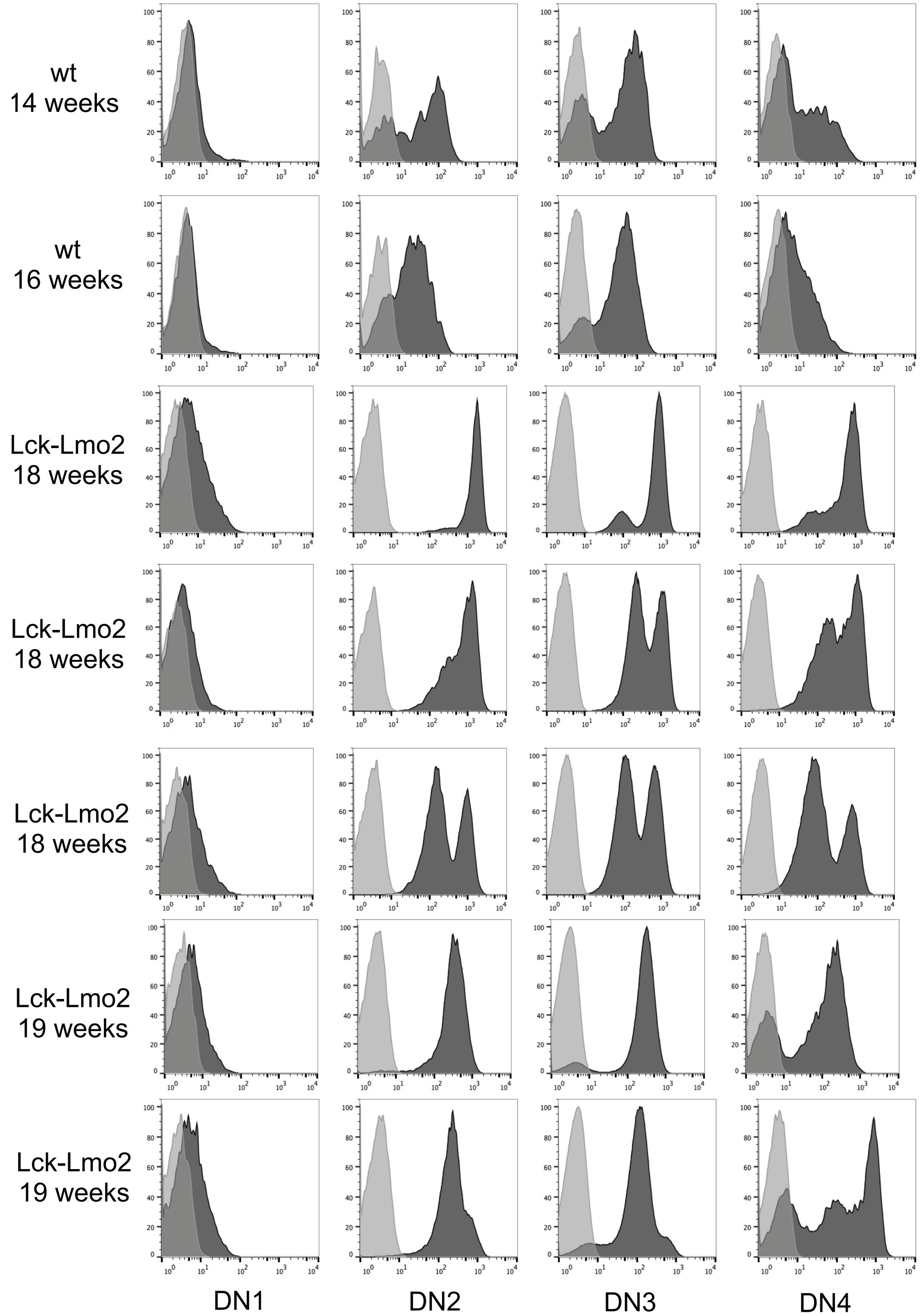

Supplementary Figure S4. Flow cytometry of CD59a expression on asymptomatic *Lck-Lmo2* mouse DN thymocytes.

Thymocytes were prepared as single cell suspensions from *Lck-Lmo2* mice or wild type equivalents and analyzed by flow cytometry with anti-CD59a antibody. Expression of CD59a was measured in two wild type (14 and 16 weeks old) or five asymptomatic *Lck-Lmo2* mice (18 or 19 weeks old). The curves show the results for CD59a-expressing cells (black) compared with the isotype control (grey) for pooled DN1, DN2, DN3 and DN4 cells. The x-axis shows fluorescence on a log scale.

Supplementary Figure S5

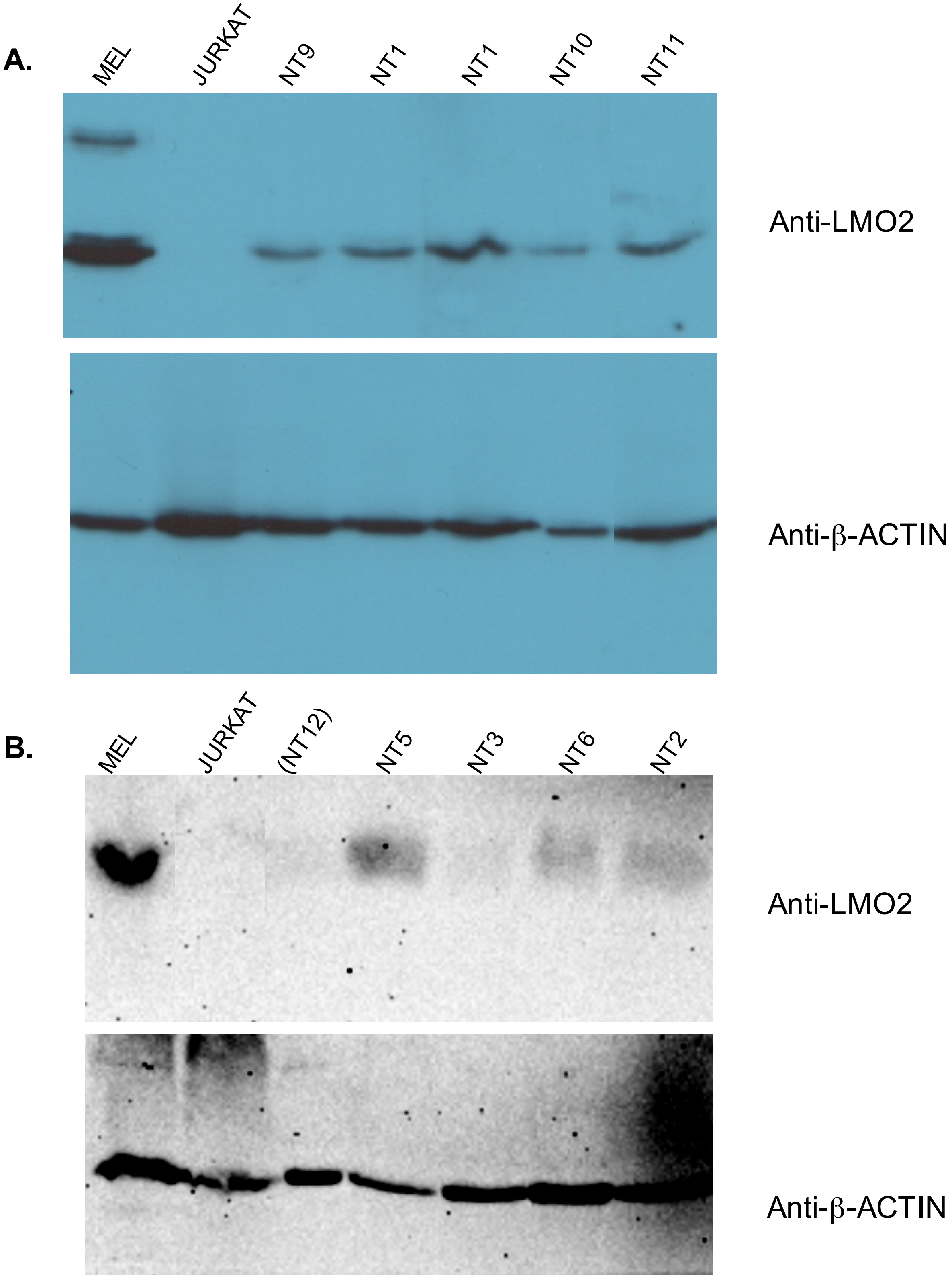

Supplementary Figure S5. Western blot analysis of LMO2 expression in human T-ALL lacking LMO2-associated translocations

1x 10^5 primary human T-ALL cells were lysed in SDS-PAGE loading buffer, boiled for 5 mins with 10mM b-mercaptoethanol and fractionated on to 15% SDS-PAGE. The gel was blotted on to nylon membranes which were incubated with mouse anti-human LMO2 antibody and signal detected using ECL methods with anti-mouse HRP for development. Anti-b-ACTIN antibody was applied to the mebranes as protein loading controls.

Supplementary Figure S6

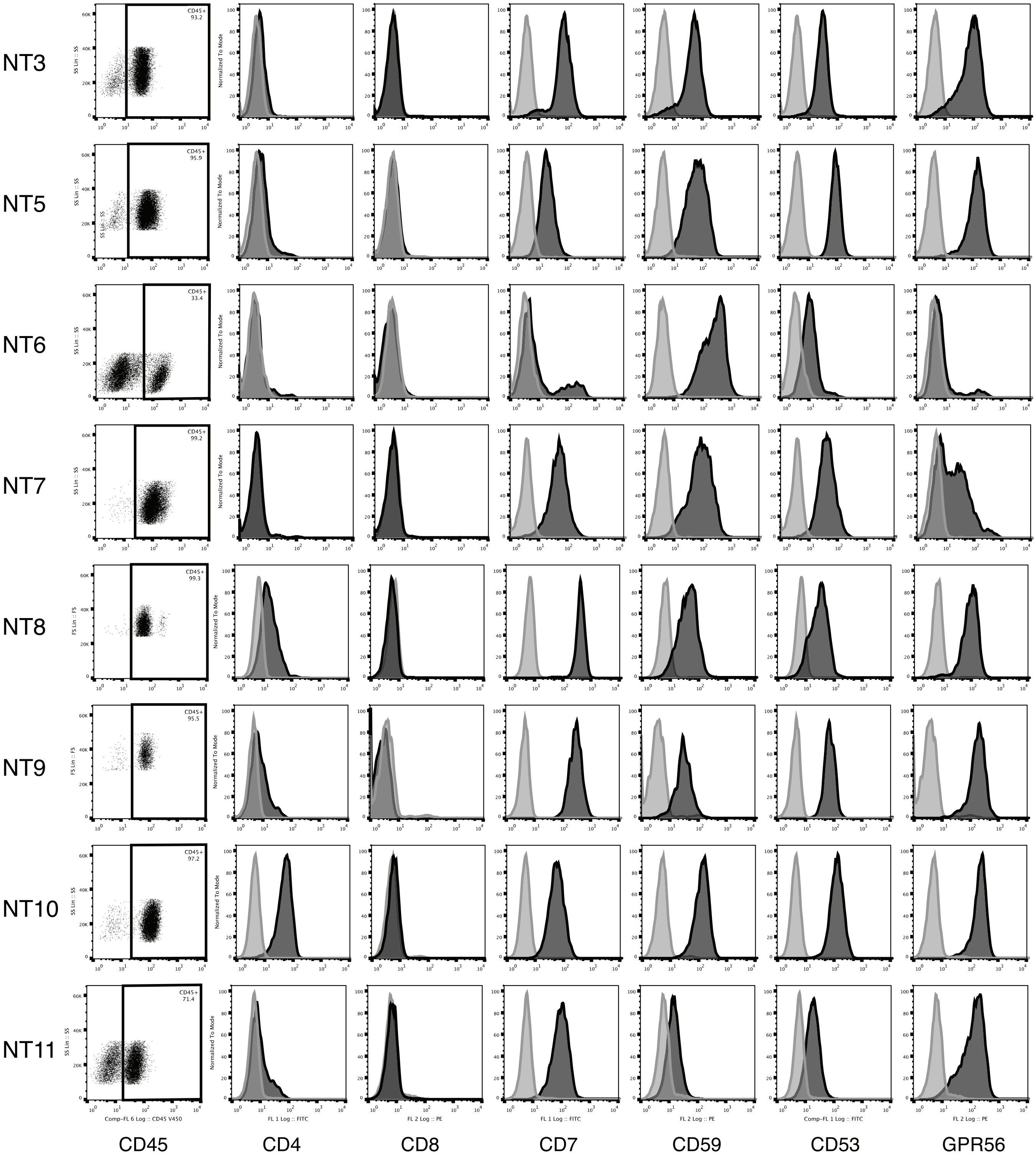

Supplementary Figure S6. Surface expression of identified markers on primary human leukaemia samples.

Eight human T-ALL samples (NT3-NT11, lacking LMO2 chromosomal translocations) were analysed for cell surface marker expression. The left hand column of dot plots shows percentage of CD45+ cells present in each leukaemic sample. All the other antibody staining are gated on the CD45+ population and each histogram shows the result for CD45+ cells on stained cells (dark grey) and cells unstained or stained with isotype control (light grey).

The x axes shows the fluorescence in log scale, the y axes represent count in cell number (scaled as a percentage of maximum count).

Supplementary Figure S7

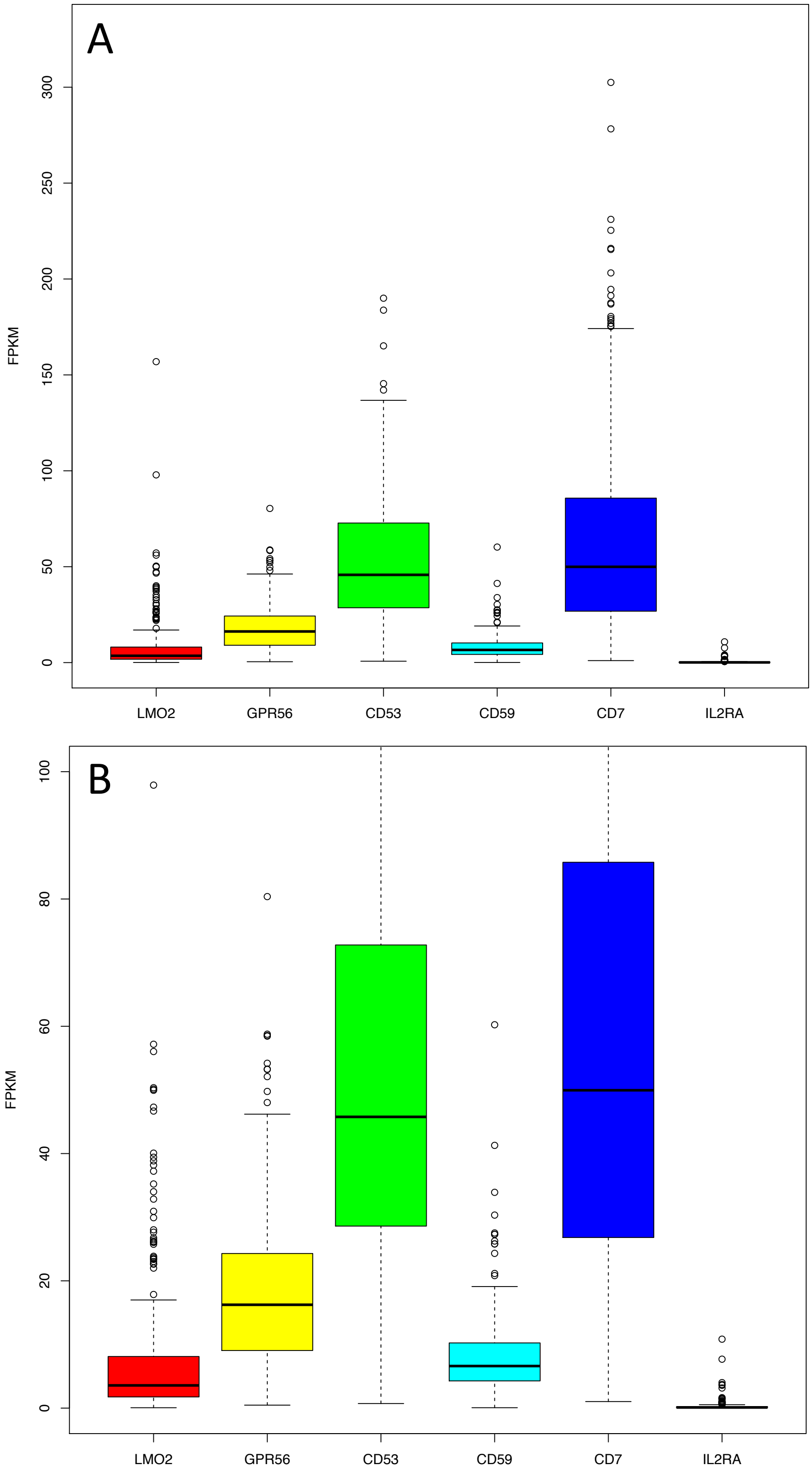

Supplementary Figure S7. The boxplots show the overall range and distribution of the gene expression levels (FPKM) of LMO2, GPR56, CD53, CD59, CD7 and IL2RA in 264 pediatric and young adult T-ALL samples. Panel A: the y axis is the FPKM values from 0 to 300 Panel B: the y axis is expanded FPKM values from 0 to 100
The data are from Liu Y et. al. The genomic landscape of pediatric and young adult T-lineage acute lymphoblastic leukemia, Nature Genetics,49(8):1211-1218 (2017)).

Supplementary Figure S8

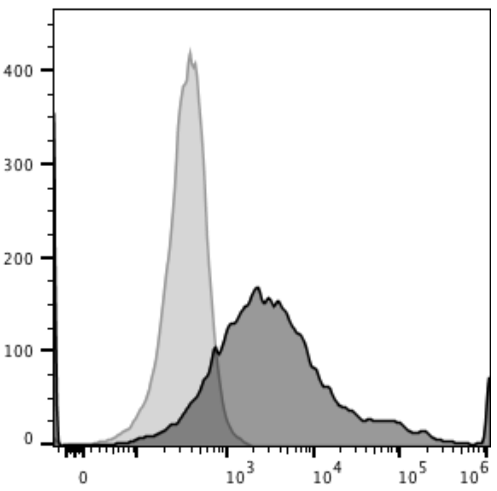

Supplementary Figure S8. Surface expression of B2AR in reporter HEK293T cells
5xGAL4UAS-luciferase reporter HEK293T cells were transfected with the B2AR-NUB plasmid. Surface expression was analysed 48 hours post-transfection by FACS using a PE B2AR (R11E1) antibody. The histogram shows the B2AR+ population (dark grey), as compared to the unstained population (light grey). The x axes shows the fluorescence in log scale and the y axes represents the cell count.

Supplementary Figure S9

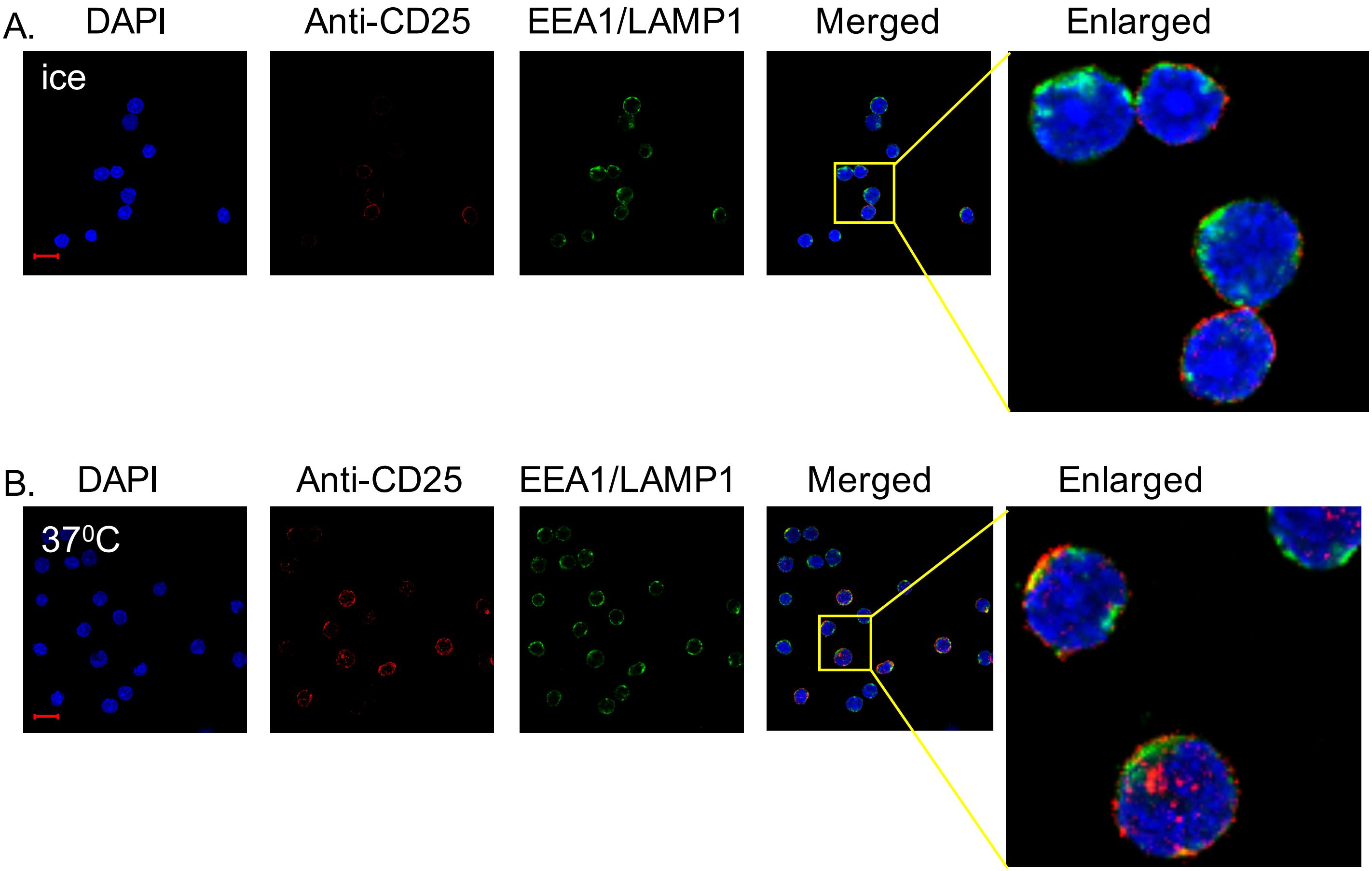

Supplementary Figure S9. CD25 expression on thymocytes from Lck-Lmo2 mouse
A 28-week-old, female Lck-Lmo2 transgenic mouse thymus was removed and a single suspension prepared. The cells were stained with anti-CD25 antibody coupled to APC (red), with anti-EEA1 and anti-LAMP1 followed by anti-rabbit A488 secondary antibody (green), and with DAPI (blue). The cells were analyzed by confocal microscopy and images shown at x63 magnification except the right-hand panels were enlarged as indicated.
A. Five panels showing cells stained with antibody & incubated for 2.5 hours on ice.
B. Five panels showing cells stained with antibody & incubated for 30minutes on ice and then 2 hours at 37^0C
The scale bar of 10μm is shown on the DAPI stained images.

Supplementary Figure S10. Sequence of surface marker cDNAs used for GFP complementation

DNA and amino acid sequences of the constructs used in the GFP complementation assay, internalisation (A-F) and MaMTH experiments are shown (G-H). In A-F the Gly-Ser linker regions between the antigen and GFP10/11 are shown in pink and the GFP10 and GFP11 sequences are shown in green and blue, respectively. The pEF-BOS expression vector (Invitrogen) was used. For the B2AR-NUB sequence the Gly-Ser linker region is shown in pink, the FLAG-tag in green and the NUB domain in blue (G). For the CD25-CUB-TF sequence the Gly-Ser linker region is shown in pink, the CUB domain in blue, the spacer in green and the GAL4 TF region in orange (H).

A. Sequence of GPR56-10
B. Sequence of CD7-10
C. Sequence of CD53-10
D. Sequence of CD25-11
E. Sequence of GFP 1-9
F. Sequence of B2AR-10
G. Sequence of B2AR-NUB
H. Sequence of CD25-CUB-TF

A. Sequence of pEF-GPR56-G4S)3-GFP10

```
        10        20        30        40        50        60
CACGTGATGACTCCCCAGTCGCTGCTGCAGACGACACTGTTCCTGCTGAGTCTGCTCTTC
GTGCACTACTGAGGGGTCAGCGACGACGTCTGCTGTGACAAGGACGACTCAGACGAGAAG
       M  T  P  Q  S  L  L  Q  T  T  L  F  L  L  S  L  L  F

        70        80        90       100       110       120
CTGGTCCAAGGTGCCCACGGCAGGGGCCACAGGGAAGACTTTCGCTTCTGCAGCCAGCGG
GACCAGGTTCCACGGGTGCCGTCCCCGGTGTCCCTTCTGAAAGCGAAGACGTCGGTCGCC
 L  V  Q  G  A  H  G  R  G  H  R  E  D  F  R  F  C  S  Q  R

       130       140       150       160       170       180
AACCAGACACACAGGAGCAGCCTCCACTACAAACCCACACCAGACCTGCGCATCTCCATC
TTGGTCTGTGTGTCCTCGTCGGAGGTGATGTTTGGGTGTGGTCTGGACGCGTAGAGGTAG
 N  Q  T  H  R  S  S  L  H  Y  K  P  T  P  D  L  R  I  S  I

       190       200       210       220       230       240
GAGAACTCCGAAGAGGCCCTCACAGTCCATGCCCCTTTCCCTGCAGCCCACCCTGCTTCC
CTCTTGAGGCTTCTCCGGGAGTGTCAGGTACGGGGAAAGGGACGTCGGGTGGGACGAAGG
 E  N  S  E  E  A  L  T  V  H  A  P  F  P  A  A  H  P  A  S

       250       260       270       280       290       300
CGATCCTTCCCTGACCCCAGGGGCCTCTACCACTTCTGCCTCTACTGGAACCGACATGCT
GCTAGGAAGGGACTGGGGTCCCCGGAGATGGTGAAGACGGAGATGACCTTGGCTGTACGA
 R  S  F  P  D  P  R  G  L  Y  H  F  C  L  Y  W  N  R  H  A

       310       320       330       340       350       360
GGGAGATTACATCTTCTCTATGGCAAGCGTGACTTCTTGCTGAGTGACAAAGCCTCTAGC
CCCTCTAATGTAGAAGAGATACCGTTCGCACTGAAGAACGACTCACTGTTTCGGAGATCG
 G  R  L  H  L  L  Y  G  K  R  D  F  L  L  S  D  K  A  S  S

       370       380       390       400       410       420
CTCCTCTGCTTCCAGCACCAGGAGGAGAGCCTGGCTCAGGGCCCCCCGCTGTTAGCCACT
GAGGAGACGAAGGTCGTGGTCCTCCTCTCGGACCGAGTCCCGGGGGGCGACAATCGGTGA
 L  L  C  F  Q  H  Q  E  E  S  L  A  Q  G  P  P  L  L  A  T

       430       440       450       460       470       480
TCTGTCACCTCCTGGTGGAGCCCTCAGAACATCAGCCTGCCCAGTGCCGCCAGCTTCACC
AGACAGTGGAGGACCACCTCGGGAGTCTTGTAGTCGGACGGGTCACGGCGGTCGAAGTGG
 S  V  T  S  W  W  S  P  Q  N  I  S  L  P  S  A  A  S  F  T

       490       500       510       520       530       540
TTCTCCTTCCACAGTCCTCCCCACACGGCCGCTCACAATGCCTCGGTGGACATGTGCGAG
AAGAGGAAGGTGTCAGGAGGGGTGTGCCGGCGAGTGTTACGGAGCCACCTGTACACGCTC
 F  S  F  H  S  P  P  H  T  A  A  H  N  A  S  V  D  M  C  E
```

```
       550       560       570       580       590       600
CTCAAAAGGGACCTCCAGCTGCTCAGCCAGTTCCTGAAGCATCCCCAGAAGGCCTCAAGG
GAGTTTTCCCTGGAGGTCGACGAGTCGGTCAAGGACTTCGTAGGGGTCTTCCGGAGTTCC
 L  K  R  D  L  Q  L  L  S  Q  F  L  K  H  P  Q  K  A  S  R

       610       620       630       640       650       660
AGGCCCTCGGCTGCCCCCGCCAGCCAGCAGTTGCAGAGCCTGGAGTCGAAACTGACCTCT
TCCGGGAGCCGACGGGGGCGGTCGGTCGTCAACGTCTCGGACCTCAGCTTTGACTGGAGA
 R  P  S  A  A  P  A  S  Q  Q  L  Q  S  L  E  S  K  L  T  S

       670       680       690       700       710       720
GTGAGATTCATGGGGGACATGGTGTCCTTCGAGGAGGACCGGATCAACGCCACGGTGTGG
CACTCTAAGTACCCCCTGTACCACAGGAAGCTCCTCCTGGCCTAGTTGCGGTGCCACACC
 V  R  F  M  G  D  M  V  S  F  E  E  D  R  I  N  A  T  V  W

       730       740       750       760       770       780
AAGCTCCAGCCCACAGCCGGCCTCCAGGACCTGCACATCCACTCCCAGCAGGAGGAGGAG
TTCGAGGTCGGGTGTCGGCCGGAGGTCCTGGACGTGTAGGTGAGGGTCGTCCTCCTCCTC
 K  L  Q  P  T  A  G  L  Q  D  L  H  I  H  S  Q  Q  E  E  E

       790       800       810       820       830       840
CAGAGCGAGATCATGGAGTACTCGGTGCTGCTGCCTCGAACACTCTTCCAGAGGACGAAA
GTCTCGCTCTAGTACCTCATGAGCCACGACGACGGAGCTTGTGAGAAGGTCTCCTGCTTT
 Q  S  E  I  M  E  Y  S  V  L  L  P  R  T  L  F  Q  R  T  K

       850       860       870       880       890       900
GGCCGGAGCGGGGAGGCTGAGAAGAGACTCCTCCTGGTGGACTTCAGCAGCCAAGCCCTG
CCGGCCTCGCCCCTCCGACTCTTCTCTGAGGAGGACCACCTGAAGTCGTCGGTTCGGGAC
 G  R  S  G  E  A  E  K  R  L  L  L  V  D  F  S  S  Q  A  L

       910       920       930       940       950       960
TTCCAGGACAAGAACTCCAGCCAAGTCCTGGGTGAGAAGGTCTTGGGGATTGTGGTACAG
AAGGTCCTGTTCTTGAGGTCGGTTCAGGACCCACTCTTCCAGAACCCCTAACACCATGTC
 F  Q  D  K  N  S  S  Q  V  L  G  E  K  V  L  G  I  V  V  Q

       970       980       990      1000      1010      1020
AACACCAAAGTAGCCAACCTCACGGAGCCCGTGGTGCTCACCTTCCAGCACCAGCTACAG
TTGTGGTTTCATCGGTTGGAGTGCCTCGGGCACCACGAGTGGAAGGTCGTGGTCGATGTC
 N  T  K  V  A  N  L  T  E  P  V  V  L  T  F  Q  H  Q  L  Q

      1030      1040      1050      1060      1070      1080
CCGAAGAATGTGACTCTGCAATGTGTGTTCTGGGTTGAAGACCCCACATTGAGCAGCCCG
GGCTTCTTACACTGAGACGTTACACACAAGACCCAACTTCTGGGGTGTAACTCGTCGGGC
 P  K  N  V  T  L  Q  C  V  F  W  V  E  D  P  T  L  S  S  P

      1090      1100      1110      1120      1130      1140
GGGCATTGGAGCAGTGCTGGGTGTGAGACCGTCAGGAGAGAAACCCAAACATCCTGCTTC
CCCGTAACCTCGTCACGACCCACACTCTGGCAGTCCTCTCTTTGGGTTTGTAGGACGAAG
 G  H  W  S  S  A  G  C  E  T  V  R  R  E  T  Q  T  S  C  F

      1150      1160      1170      1180      1190      1200
TGCAACCACTTGACCTACTTTGCAGTGTTGATGGTCTCCTCGGTGGAGGTGGACGCCGTG
ACGTTGGTGAACTGGATGAAACGTCACAACTACCAGAGGAGCCACCTCCACCTGCGGCAC
 C  N  H  L  T  Y  F  A  V  L  M  V  S  S  V  E  V  D  A  V

      1210      1220      1230      1240      1250      1260
CACAAGCACTACCTGAGCCTCCTCTCCTACGTGGGCTGTGTCGTCTCTGCCCTGGCCTGC
GTGTTCGTGATGGACTCGGAGGAGAGGATGCACCCGACACAGCAGAGACGGGACCGGACG
 H  K  H  Y  L  S  L  L  S  Y  V  G  C  V  V  S  A  L  A  C

      1270      1280      1290      1300      1310      1320
CTTGTCACCATTGCCGCCTACCTCTGCTCCAGGGTGCCCCTGCCGTGCAGGAGGAAACCT
GAACAGTGGTAACGGCGGATGGAGACGAGGTCCCACGGGGACGGCACGTCCTCCTTTGGA
 L  V  T  I  A  A  Y  L  C  S  R  V  P  L  P  C  R  R  K  P

      1330      1340      1350      1360      1370      1380
CGGGACTACACCATCAAGGTGCACATGAACCTGCTGCTGGCCGTCTTCCTGCTGGACACG
GCCCTGATGTGGTAGTTCCACGTGTACTTGGACGACGACCGGCAGAAGGACGACCTGTGC
 R  D  Y  T  I  K  V  H  M  N  L  L  L  A  V  F  L  L  D  T>

      1390      1400      1410      1420      1430      1440
AGCTTCCTGCTCAGCGAGCCGGTGGCCCTGACAGGCTCTGAGGCTGGCTGCCGAGCCAGT
TCGAAGGACGAGTCGCTCGGCCACCGGGACTGTCCGAGACTCCGACCGACGGCTCGGTCA
 S  F  L  L  S  E  P  V  A  L  T  G  S  E  A  G  C  R  A  S
```

```
      1450      1460      1470      1480      1490      1500
GCCATCTTCCTGCACTTCTCCCTGCTCACCTGCCTTTCCTGGATGGGCCTCGAGGGGTAC
CGGTAGAAGGACGTGAAGAGGGACGAGTGGACGGAAAGGACCTACCCGGAGCTCCCCATG
 A  I  F  L  H  F  S  L  L  T  C  L  S  W  M  G  L  E  G  Y

      1510      1520      1530      1540      1550      1560
AACCTCTACCGACTCGTGGTGGAGGTCTTTGGCACCTATGTCCCTGGCTACCTACTCAAG
TTGGAGATGGCTGAGCACCACCTCCAGAAACCGTGGATACAGGGACCGATGGATGAGTTC
 N  L  Y  R  L  V  V  E  V  F  G  T  Y  V  P  G  Y  L  L  K

      1570      1580      1590      1600      1610      1620
CTGAGCGCCATGGGCTGGGGCTTCCCCATCTTTCTGGTGACGCTGGTGGCCCTGGTGGAT
GACTCGCGGTACCCGACCCCGAAGGGGTAGAAAGACCACTGCGACCACCGGGACCACCTA
 L  S  A  M  G  W  G  F  P  I  F  L  V  T  L  V  A  L  V  D>

      1630      1640      1650      1660      1670      1680
GTGGACAACTATGGCCCCATCATCTTGGCTGTGCATAGGACTCCAGAGGGCGTCATCTAC
CACCTGTTGATACCGGGGTAGTAGAACCGACACGTATCCTGAGGTCTCCCGCAGTAGATG
 V  D  N  Y  G  P  I  I  L  A  V  H  R  T  P  E  G  V  I  Y

      1690      1700      1710      1720      1730      1740
CCTTCCATGTGCTGGATACGGGACTCCCTGGTCAGCTACATCACCAACCTGGGCCTCTTC
GGAAGGTACACGACCTATGCCCTGAGGGACCAGTCGATGTAGTGGTTGGACCCGGAGAAG
 P  S  M  C  W  I  R  D  S  L  V  S  Y  I  T  N  L  G  L  F

      1750      1760      1770      1780      1790      1800
AGCCTGGTGTTTCTGTTCAACATGGCCATGCTAGCCACCATGGTGGTGCAGATCCTGCGG
TCGGACCACAAAGACAAGTTGTACCGGTACGATCGGTGGTACCACCACGTCTAGGACGCC
 S  L  V  F  L  F  N  M  A  M  L  A  T  M  V  V  Q  I  L  R

      1810      1820      1830      1840      1850      1860
CTGCGCCCCCACACCCAAAAGTGGTCACATGTGCTGACACTGCTGGGCCTCAGCCTGGTC
GACGCGGGGGTGTGGGTTTTCACCAGTGTACACGACTGTGACGACCCGGAGTCGGACCAG
 L  R  P  H  T  Q  K  W  S  H  V  L  T  L  L  G  L  S  L  V

      1870      1880      1890      1900      1910      1920
CTTGGCCTGCCCTGGGCCTTGATCTTCTTCTCCTTTGCTTCTGGCACCTTCCAGCTTGTC
GAACCGGACGGGACCCGGAACTAGAAGAAGAGGAAACGAAGACCGTGGAAGGTCGAACAG
 L  G  L  P  W  A  L  I  F  F  S  F  A  S  G  T  F  Q  L  V

      1930      1940      1950      1960      1970      1980
GTCCTCTACCTTTTCAGCATCATCACCTCCTTCCAAGGCTTCCTCATCTTCATCTGGTAC
CAGGAGATGGAAAAGTCGTAGTAGTGGAGGAAGGTTCCGAAGGAGTAGAAGTAGACCATG
 V  L  Y  L  F  S  I  I  T  S  F  Q  G  F  L  I  F  I  W  Y

      1990      2000      2010      2020      2030      2040
TGGTCCATGCGGCTGCAGGCCCGGGGTGGCCCCTCCCCTCTGAAGAGCAACTCAGACAGC
ACCAGGTACGCCGACGTCCGGGCCCCACCGGGGAGGGGAGACTTCTCGTTGAGTCTGTCG
 W  S  M  R  L  Q  A  R  G  G  P  S  P  L  K  S  N  S  D  S

      2050      2060      2070      2080      2090      2100
GCCAGGCTCCCCATCAGCTCGGGCAGCACCTCGTCCAGCCGCATCCATATGGGCGGCGGA
CGGTCCGAGGGGTAGTCGAGCCCGTCGTGGAGCAGGTCGGCGTAGGTATACCCGCCGCCT
 A  R  L  P  I  S  S  G  S  T  S  S  S  R  I  H  M  G  G  G

      2110      2120      2130      2140      2150      2160
GGATCTGGGGGCGGAGGAAGTGGGGGAGGGGGCTCTCTCGAGGACCTGCCTGATGATCAC
CCTAGACCCCCGCCTCCTTCACCCCCTCCCCCGAGAGAGCTCCTGGACGGACTACTAGTG
 G  S  G  G  G  G  S  G  G  G  G  S  L  E  D  L  P  D  D  H

          2170      2180      2190      2200      2210
TATCTGTCTACACAGACAATCCTGTCTAAGGATCTGAATGGGACCGACGTGTAA
ATAGACAGATGTGTCTGTTAGGACAGATTCCTAGACTTACCCTGGCTGCACATT
 Y  L  S  T  Q  T  I  L  S  K  D  L  N  G  T  D  V  *
```

B. Sequence of pEF-CD7-(G4S)3-GFP10

```
        10        20        30        40        50        60
CACGTGATGGCCGGGCCTCCGAGGCTCCTGCTGCTGCCCCTGCTTCTGGCGCTGGCTCGC
GTGCACTACCGGCCCGGAGGCTCCGAGGACGACGACGGGGACGAAGACCGCGACCGAGCG
       M  A  G  P  P  R  L  L  L  L  P  L  L  L  A  L  A  R

        70        80        90       100       110       120
GGCCTGCCTGGGGCCCTGGCTGCCCAAGAGGTGCAGCAGTCTCCCCACTGCACGACTGTC
CCGGACGGACCCCGGGACCGACGGGTTCTCCACGTCGTCAGAGGGGTGACGTGCTGACAG
 G  L  P  G  A  L  A  A  Q  E  V  Q  Q  S  P  H  C  T  T  V
```

```
       130       140       150       160       170       180
CCCGTGGGAGCCTCCGTCAACATCACCTGCTCCACCAGCGGGGGCCTGCGTGGGATCTAC
GGGCACCCTCGGAGGCAGTTGTAGTGGACGAGGTGGTCGCCCCCGGACGCACCCTAGATG
 P  V  G  A  S  V  N  I  T  C  S  T  S  G  G  L  R  G  I  Y

       190       200       210       220       230       240
CTGAGGCAGCTCGGGCCACAGCCCCAAGACATCATTTACTACGAGGACGGGGTGGTGCCC
GACTCCGTCGAGCCCGGTGTCGGGGTTCTGTAGTAAATGATGCTCCTGCCCCACCACGGG
 L  R  Q  L  G  P  Q  P  Q  D  I  I  Y  Y  E  D  G  V  V  P

       250       260       270       280       290       300
ACTACGGACAGACGGTTCCGGGGCCGCATCGACTTCTCAGGGTCCCAGGACAACCTGACT
TGATGCCTGTCTGCCAAGGCCCCGGCGTAGCTGAAGAGTCCCAGGGTCCTGTTGGACTGA
 T  T  D  R  R  F  R  G  R  I  D  F  S  G  S  Q  D  N  L  T

       310       320       330       340       350       360
ATCACCATGCACCGCCTGCAGCTGTCGGACACTGGCACCTACACCTGCCAGGCCATCACG
TAGTGGTACGTGGCGGACGTCGACAGCCTGTGACCGTGGATGTGGACGGTCCGGTAGTGC
 I  T  M  H  R  L  Q  L  S  D  T  G  T  Y  T  C  Q  A  I  T

       370       380       390       400       410       420
GAGGTCAATGTCTACGGCTCCGGCACCCTGGTCCTGGTGACAGAGGAACAGTCCCAAGGA
CTCCAGTTACAGATGCCGAGGCCGTGGGACCAGGACCACTGTCTCCTTGTCAGGGTTCCT
 E  V  N  V  Y  G  S  G  T  L  V  L  V  T  E  E  Q  S  Q  G

       430       440       450       460       470       480
TGGCACAGATGCTCGGACGCCCCACCAAGGGCCTCTGCCCTCCCTGCCCCACCGACAGGC
ACCGTGTCTACGAGCCTGCGGGGTGGTTCCCGGAGACGGGAGGGACGGGGTGGCTGTCCG
 W  H  R  C  S  D  A  P  P  R  A  S  A  L  P  A  P  P  T  G

       490       500       510       520       530       540
TCCGCCCTCCCTGACCCGCAGACAGCCTCTGCCCTCCCTGACCCGCCAGCAGCCTCTGCC
AGGCGGGAGGGACTGGGCGTCTGTCGGAGACGGGAGGGACTGGGCGGTCGTCGGAGACGG
 S  A  L  P  D  P  Q  T  A  S  A  L  P  D  P  P  A  A  S  A

       550       560       570       580       590       600
CTCCCTGCGGCCCTGGCGGTGATCTCCTTCCTCCTCGGGCTGGGCCTGGGGGTGGCGTGT
GAGGGACGCCGGGACCGCCACTAGAGGAAGGAGGAGCCCGACCCGGACCCCCACCGCACA
 L  P  A  A  L  A  V  I  S  F  L  L  G  L  G  L  G  V  A  C

       610       620       630       640       650       660
GTGCTGGCGAGGACACAGATAAAGAAACTGTGCTCGTGGCGGGATAAGAACTCGGCGGCA
CACGACCGCTCCTGTGTCTATTTCTTTGACACGAGCACCGCCCTATTCTTGAGCCGCCGT
 V  L  A  R  T  Q  I  K  K  L  C  S  W  R  D  K  N  S  A  A

       670       680       690       700       710       720
TGTGTGGTGTACGAGGACATGTCGCACAGCCGCTGCAACACGCTGTCCTCCCCCAACCAG
ACACACCACATGCTCCTGTACAGCGTGTCGGCGACGTTGTGCGACAGGAGGGGGTTGGTC
 C  V  V  Y  E  D  M  S  H  S  R  C  N  T  L  S  S  P  N  Q

       730       740       750       760       770       780
TACCAGGTCGAGGGCGGCGGAGGATCTGGGGGCGGAGGAAGTGGGGGAGGGGGCTCTTTC
ATGGTCCAGCTCCCGCCGCCTCCTAGACCCCCGCCTCCTTCACCCCCTCCCCCGAGAAAG
 Y  Q  V  E  G  G  G  G  S  G  G  G  G  S  G  G  G  G  S  F

       790       800       810       820       830       840
GAGGACCTGCCTGATGATCACTATCTGTCTACACAGACAATCCTGTCTAAGGATCTGAAT
CTCCTGGACGGACTACTAGTGATAGACAGATGTGTCTGTTAGGACAGATTCCTAGACTTA
 E  D  L  P  D  D  H  Y  L  S  T  Q  T  I  L  S  K  D  L  N

              850
GGGACCGACGTGTAA
CCCTGGCTGCACATT
 G  T  D  V  *
```

C. Sequence of pEF-CD53-(G4S)3-GFP10

```
        10        20        30        40        50        60
CACGTGATGGGCATGAGTAGCTTGAAACTGCTGAAGTATGTCCTGTTTTTCTTCAACTTG
GTGCACTACCCGTACTCATCGAACTTTGACGACTTCATACAGGACAAAAAGAAGTTGAAC
       M  G  M  S  S  L  K  L  L  K  Y  V  L  F  F  F  N  L

        70        80        90       100       110       120
CTCTTTTGGATCTGTGGCTGCTGCATTTTGGGCTTTGGGATCTACCTGCTGATCCACAAC
GAGAAAACCTAGACACCGACGACGTAAAACCCGAAACCCTAGATGGACGACTAGGTGTTG
 L  F  W  I  C  G  C  C  I  L  G  F  G  I  Y  L  L  I  H  N
```

```
        130       140       150       160       170       180
AACTTCGGAGTGCTCTTCCATAACCTCCCCTCCCTCACGCTGGGCAATGTGTTTGTCATC
TTGAAGCCTCACGAGAAGGTATTGGAGGGGAGGGAGTGCGACCCGTTACACAAACAGTAG
 N  F  G  V  L  F  H  N  L  P  S  L  T  L  G  N  V  F  V  I

        190       200       210       220       230       240
GTGGGCTCTATTATCATGGTAGTTGCCTTCCTGGGCTGCATGGGCTCTATCAAGGAAAAC
CACCCGAGATAATAGTACCATCAACGGAAGGACCCGACGTACCCGAGATAGTTCCTTTTG
 V  G  S  I  I  M  V  V  A  F  L  G  C  M  G  S  I  K  E  N>

        250       260       270       280       290       300
AAGTGTCTGCTTATGTCGTTCTTCATCCTGCTGCTGATTATCCTCCTTGCTGAGGTGACC
TTCACAGACGAATACAGCAAGAAGTAGGACGACGACTAATAGGAGGAACGACTCCACTGG
 K  C  L  L  M  S  F  F  I  L  L  L  I  I  L  L  A  E  V  T

        310       320       330       340       350       360
TTGGCCATCCTGCTCTTTGTATATGAACAGAAGCTGAATGAGTATGTGGCTAAGGGTCTG
AACCGGTAGGACGAGAAACATATACTTGTCTTCGACTTACTCATACACCGATTCCCAGAC
 L  A  I  L  L  F  V  Y  E  Q  K  L  N  E  Y  V  A  K  G  L

        370       380       390       400       410       420
ACCGACAGCATCCACCGTTACCACTCAGACAATAGCACCAAGGCAGCGTGGGACTCCATC
TGGCTGTCGTAGGTGGCAATGGTGAGTCTGTTATCGTGGTTCCGTCGCACCCTGAGGTAG
 T  D  S  I  H  R  Y  H  S  D  N  S  T  K  A  A  W  D  S  I

        430       440       450       460       470       480
CAGTCATTTCTGCAGTGTTGTGGTATAAATGGCACGAGTGATTGGACCAGTGGCCCACCA
GTCAGTAAAGACGTCACAACACCATATTTACCGTGCTCACTAACCTGGTCACCGGGTGGT
 Q  S  F  L  Q  C  C  G  I  N  G  T  S  D  W  T  S  G  P  P

        490       500       510       520       530       540
GCATCTTGCCCCTCAGATCGAAAAGTGGAGGGTTGCTATGCGAAAGCAAGACTGTGGTTT
CGTAGAACGGGGAGTCTAGCTTTTCACCTCCCAACGATACGCTTTCGTTCTGACACCAAA
 A  S  C  P  S  D  R  K  V  E  G  C  Y  A  K  A  R  L  W  F

        550       560       570       580       590       600
CATTCCAATTTCCTGTATATCGGAATCATCACCATCTGTGTATGTGTGATTGAGGTGTTG
GTAAGGTTAAAGGACATATAGCCTTAGTAGTGGTAGACACATACACACTAACTCCACAAC
 H  S  N  F  L  Y  I  G  I  I  T  I  C  V  C  V  I  E  V  L

        610       620       630       640       650       660
GGGATGTCCTTTGCACTGACCCTGAACTGCCAGATTGACAAAACCAGCCAGACCATAGGG
CCCTACAGGAAACGTGACTGGGACTTGACGGTCTAACTGTTTTGGTCGGTCTGGTATCCC
 G  M  S  F  A  L  T  L  N  C  Q  I  D  K  T  S  Q  T  I  G

        670       680       690       700       710       720
CTACATATGGGCGGCGGAGGATCTGGGGGCGGAGGAAGTGGGGGAGGGGGCTCTCTCGAG
GATGTATACCCGCCGCCTCCTAGACCCCCGCCTCCTTCACCCCCTCCCCCGAGAGAGCTC
 L  H  M  G  G  G  G  S  G  G  G  G  S  G  G  G  G  S  L  E

        730       740       750       760       770       780
GACCTGCCTGATGATCACTATCTGTCTACACAGACAATCCTGTCTAAGGATCTGAATGGG
CTGGACGGACTACTAGTGATAGACAGATGTGTCTGTTAGGACAGATTCCTAGACTTACCC
 D  L  P  D  D  H  Y  L  S  T  Q  T  I  L  S  K  D  L  N  G

          790
ACCGACGTGTAA
TGGCTGCACATT
 T  D  V  *
```

D. Sequence of pEF-CD25-(G4S)3-GFP11

```
         10        20        30        40        50        60
CACGTGATGAGTATGGATTCATACCTGCTGATGTGGGGACTGCTCACGTTCATCATGGTG
GTGCACTACTCATACCTAAGTATGGACGACTACACCCCTGACGAGTGCAAGTAGTACCAC
       M  S  M  D  S  Y  L  L  M  W  G  L  L  T  F  I  M  V

         70        80        90       100       110       120
CCTGGCTGCCAGGCAGAACTCTGTGACGATGACCCGCCAGAGATCCCACACGCCACATTC
GGACCGACGGTCCGTCTTGAGACACTGCTACTGGGCGGTCTCTAGGGTGTGCGGTGTAAG
 P  G  C  Q  A  E  L  C  D  D  D  P  P  E  I  P  H  A  T  F

        130       140       150       160       170       180
AAAGCCATGGCCTACAAGGAAGGAACCATGTTGAACTGTGAATGCAAGAGAGGTTTCCGC
TTTCGGTACCGGATGTTCCTTCCTTGGTACAACTTGACACTTACGTTCTCTCCAAAGGCG
 K  A  M  A  Y  K  E  G  T  M  L  N  C  E  C  K  R  G  F  R
```

```
       190       200       210       220       230       240
AGAATAAAAAGCGGGTCACTCTATATGCTCTGTACAGGAAACTCTAGCCACTCGTCCTGG
TCTTATTTTTCGCCCAGTGAGATATACGAGACATGTCCTTTGAGATCGGTGAGCAGGACC
 R  I  K  S  G  S  L  Y  M  L  C  T  G  N  S  S  H  S  S  W

       250       260       270       280       290       300
GACAACCAATGTCAATGCACAAGCTCTGCCACTCGGAACACAACGAAACAAGTGACACCT
CTGTTGGTTACAGTTACGTGTTCGAGACGGTGAGCCTTGTGTTGCTTTGTTCACTGTGGA
 D  N  Q  C  Q  C  T  S  S  A  T  R  N  T  T  K  Q  V  T  P

       310       320       330       340       350       360
CAACCTGAAGAACAGAAAGAAAGGAAAACCACAGAAATGCAAAGTCCAATGCAGCCAGTG
GTTGGACTTCTTGTCTTTCTTTCCTTTTGGTGTCTTTACGTTTCAGGTTACGTCGGTCAC
 Q  P  E  E  Q  K  E  R  K  T  T  E  M  Q  S  P  M  Q  P  V

       370       380       390       400       410       420
GACCAAGCGAGCCTTCCAGGTCACTGCAGGGAACCTCCACCATGGGAAAATGAAGCCACA
CTGGTTCGCTCGGAAGGTCCAGTGACGTCCCTTGGAGGTGGTACCCTTTTACTTCGGTGT
 D  Q  A  S  L  P  G  H  C  R  E  P  P  P  W  E  N  E  A  T

       430       440       450       460       470       480
GAGAGAATTTATCATTTCGTGGTGGGGCAGATGGTTTATTATCAGTGCGTCCAGGGATAC
CTCTCTTAAATAGTAAAGCACCACCCCGTCTACCAAATAATAGTCACGCAGGTCCCTATG
 E  R  I  Y  H  F  V  V  G  Q  M  V  Y  Y  Q  C  V  Q  G  Y

       490       500       510       520       530       540
AGGGCTCTACACAGAGGTCCTGCTGAGAGCGTCTGCAAAATGACCCACGGGAAGACAAGG
TCCCGAGATGTGTCTCCAGGACGACTCTCGCAGACGTTTTACTGGGTGCCCTTCTGTTCC
 R  A  L  H  R  G  P  A  E  S  V  C  K  M  T  H  G  K  T  R

       550       560       570       580       590       600
TGGACCCAGCCCCAGCTCATATGCACAGGTGAAATGGAGACCAGTCAGTTTCCAGGTGAA
ACCTGGGTCGGGGTCGAGTATACGTGTCCACTTTACCTCTGGTCAGTCAAAGGTCCACTT
 W  T  Q  P  Q  L  I  C  T  G  E  M  E  T  S  Q  F  P  G  E

       610       620       630       640       650       660
GAGAAGCCTCAGGCAAGCCCCGAAGGCCGTCCTGAGAGTGAGACTTCCTGCCTCGTCACA
CTCTTCGGAGTCCGTTCGGGGCTTCCGGCAGGACTCTCACTCTGAAGGACGGAGCAGTGT
 E  K  P  Q  A  S  P  E  G  R  P  E  S  E  T  S  C  L  V  T

       670       680       690       700       710       720
ACAACAGATTTTCAAATACAGACAGAAATGGCTGCAACCATGGAGACGTCCATATTTACA
TGTTGTCTAAAAGTTTATGTCTGTCTTTACCGACGTTGGTACCTCTGCAGGTATAAATGT
 T  T  D  F  Q  I  Q  T  E  M  A  A  T  M  E  T  S  I  F  T

       730       740       750       760       770       780
ACAGAGTACCAGGTAGCAGTGGCCGGCTGTGTTTTCCTGCTGATCAGCGTCCTCCTCCTG
TGTCTCATGGTCCATCGTCACCGGCCGACACAAAAGGACGACTAGTCGCAGGAGGAGGAC
 T  E  Y  Q  V  A  V  A  G  C  V  F  L  L  I  S  V  L  L  L

       790       800       810       820       830       840
AGTGGGCTCACCTGGCAGCGGAGACAGAGGAAGAGTAGAAGAACAATCTCCAGAGGCGGC
TCACCCGAGTGGACCGTCGCCTCTGTCTCCTTCTCATCTTCTTGTTAGAGGTCTCCGCCG
 S  G  L  T  W  Q  R  R  Q  R  K  S  R  R  T  I  S  R  G  G

       850       860       870       880       890       900
GGAGGATCTGGGGGCGGAGGAAGTGGGGGAGGGGGCTCTGCGGCCGCCACCAGCGAGAAG
CCTCCTAGACCCCCGCCTCCTTCACCCCCTCCCCCGAGACGCCGGCGGTGGTCGCTCTTC
 G  G  S  G  G  G  G  S  G  G  G  G  S  A  A  A  T  S  E  K

       910       920       930       940       950       960
CGGGATCATATGGTGCTGCTGGAATACGTGACCGCCGCTGGCATCACCGACGCCAGCTAA
GCCCTAGTATACCACGACGACCTTATGCACTGGCGGCGACCGTAGTGGCTGCGGTCGATT
 R  D  H  M  V  L  L  E  Y  V  T  A  A  G  I  T  D  A  S  *
```

E. Sequence of pEF-GFP1-9

```
        10        20        30        40        50        60
CACGTGATGAGGAAGGGCGAGGAACTGTTCACCGGCGTGGTGCCCATCCTGATCGAGCTG
GTGCACTACTCCTTCCCGCTCCTTGACAAGTGGCCGCACCACGGGTAGGACTAGCTCGAC
       M  R  K  G  E  E  L  F  T  G  V  V  P  I  L  I  E  L

        70        80        90       100       110       120
GATGGCGACGTGAACGGCCACAAGTTCTTCGTGCGGGGAGAGGGCGAAGGCGACGCCACA
CTACCGCTGCACTTGCCGGTGTTCAAGAAGCACGCCCCTCTCCCGCTTCCGCTGCGGTGT
 D  G  D  V  N  G  H  K  F  F  V  R  G  E  G  E  G  D  A  T
```

```
       130       140       150       160       170       180
ATCGGAAAGCTGAGCCTGAAGTTCATCTGCACCACCGGCAAGCTGCCCGTGCCTTGGCCT
TAGCCTTTCGACTCGGACTTCAAGTAGACGTGGTGGCCGTTCGACGGGCACGGAACCGGA
 I  G  K  L  S  L  K  F  I  C  T  T  G  K  L  P  V  P  W  P

       190       200       210       220       230       240
ACCCTCGTGACCACACTGACCTACGGCGTGCAGTGCTTCAGCAGATACCCCGACCATATG
TGGGAGCACTGGTGTGACTGGATGCCGCACGTCACGAAGTCGTCTATGGGGCTGGTATAC
 T  L  V  T  T  L  T  Y  G  V  Q  C  F  S  R  Y  P  D  H  M

       250       260       270       280       290       300
AAGCGGCACGACTTCTTCAAGAGCGCCATGCCCGAGGGCTACGTGCAGGAACGGACCATC
TTCGCCGTGCTGAAGAAGTTCTCGCGGTACGGGCTCCCGATGCACGTCCTTGCCTGGTAG
 K  R  H  D  F  F  K  S  A  M  P  E  G  Y  V  Q  E  R  T  I

       310       320       330       340       350       360
TACTTCAAGGACGACGGCACCTACAAGACCAGAGCCGAAGTGAAGTTCGAGGGCGACACC
ATGAAGTTCCTGCTGCCGTGGATGTTCTGGTCTCGGCTTCACTTCAAGCTCCCGCTGTGG
 Y  F  K  D  D  G  T  Y  K  T  R  A  E  V  K  F  E  G  D  T

       370       380       390       400       410       420
CTCGTGAACCGGATCGAGCTGAAGGGCATCGACTTCAAAGAGGATGGCAACATCCTGGGC
GAGCACTTGGCCTAGCTCGACTTCCCGTAGCTGAAGTTTCTCCTACCGTTGTAGGACCCG
 L  V  N  R  I  E  L  K  G  I  D  F  K  E  D  G  N  I  L  G

       430       440       450       460       470       480
CACAAGCTGGAGTACAACTTCAACAGCCACAAGGTGTACATCACCGCCGACAAGCAGAAC
GTGTTCGACCTCATGTTGAAGTTGTCGGTGTTCCACATGTAGTGGCGGCTGTTCGTCTTG
 H  K  L  E  Y  N  F  N  S  H  K  V  Y  I  T  A  D  K  Q  N

       490       500       510       520       530       540
AACGGCATCAAGGCCAACTTCACCATCCGGCACAACGTGGAAGATGGCAGCGTGCAGCTG
TTGCCGTAGTTCCGGTTGAAGTGGTAGGCCGTGTTGCACCTTCTACCGTCGCACGTCGAC
 N  G  I  K  A  N  F  T  I  R  H  N  V  E  D  G  S  V  Q  L

              550       560       570       580       590
GCCGACCACTACCAGCAGAACACCCCCATCGGAGATGGCCCCGTGCTGCTGCCTTAA
CGGCTGGTGATGGTCGTCTTGTGGGGGTAGCCTCTACCGGGGCACGACGACGGAATT
 A  D  H  Y  Q  Q  N  T  P  I  G  D  G  P  V  L  L  P  *
```

F. pEF-B2AR-(G4S)3-GFP10

```
10        20        30        40        50        60
ATGGGGCAACCCGGGAACGGCAGCGCCTTCTTGCTGGCACCCAATGGAAGCCATGCGCCG
TACCCCGTTGGGCCCTTGCCGTCGCGGAAGAACGACCGTGGGTTACCTTCGGTACGCGGC
M  G  Q  P  G  N  G  S  A  F  L  L  A  P  N  G  S  H  A  P

70        80        90        100       110       120
GACCACGACGTCACGCAGGAAAGGGACGAGGTGTGGGTGGTGGGCATGGGCATCGTCATG
CTGGTGCTGCAGTGCGTCCTTTCCCTGCTCCACACCCACCACCCGTACCCGTAGCAGTAC
D  H  D  V  T  Q  E  R  D  E  V  W  V  V  G  M  G  I  V  M

130       140       150       160       170       180
TCTCTCATCGTCCTGGCCATCGTGTTTGGCAATGTGCTGGTCATCACAGCCATTGCCAAG
AGAGAGTAGCAGGACCGGTAGCACAAACCGTTACACGACCAGTAGTGTCGGTAACGGTTC
S  L  I  V  L  A  I  V  F  G  N  V  L  V  I  T  A  I  A  K

190       200       210       220       230       240
TTCGAGCGTCTGCAGACGGTCACCAACTACTTCATCACTTCACTGGCCTGTGCTGATCTG
AAGCTCGCAGACGTCTGCCAGTGGTTGATGAAGTAGTGAAGTGACCGGACACGACTAGAC
F  E  R  L  Q  T  V  T  N  Y  F  I  T  S  L  A  C  A  D  L

250       260       270       280       290       300
GTCATGGGCCTGGCAGTGGTGCCCTTTGGGGCCGCCCATATTCTTATGAAAATGTGGACT
CAGTACCCGGACCGTCACCACGGGAAACCCCGGCGGGTATAAGAATACTTTTACACCTGA
V  M  G  L  A  V  V  P  F  G  A  A  H  I  L  M  K  M  W  T

310       320       330       340       350       360
TTTGGCAACTTCTGGTGCGAGTTTTGGACTTCCATTGATGTGCTGTGCGTCACGGCCAGC
AAACCGTTGAAGACCACGCTCAAAACCTGAAGGTAACTACACGACACGCAGTGCCGGTCG
F  G  N  F  W  C  E  F  W  T  S  I  D  V  L  C  V  T  A  S

370       380       390       400       410       420
ATTGAGACCCTGTGCGTGATCGCAGTGGATCGCTACTTTGCCATTACTTCACCTTTCAAG
TAACTCTGGGACACGCACTAGCGTCACCTAGCGATGAAACGGTAATGAAGTGGAAAGTTC
I  E  T  L  C  V  I  A  V  D  R  Y  F  A  I  T  S  P  F  K
```

```
430       440       450       460       470       480
TACCAGAGCCTGCTGACCAAGAATAAGGCCCGGGTGATCATTCTGATGGTGTGGATTGTG
ATGGTCTCGGACGACTGGTTCTTATTCCGGGCCCACTAGTAAGACTACCACACCTAACAC
Y  Q  S  L  L  T  K  N  K  A  R  V  I  I  L  M  V  W  I  V

490       500       510       520       530       540
TCAGGCCTTACCTCCTTCTTGCCCATTCAGATGCACTGGTACCGGGCCACCCACCAGGAA
AGTCCGGAATGGAGGAAGAACGGGTAAGTCTACGTGACCATGGCCCGGTGGGTGGTCCTT
S  G  L  T  S  F  L  P  I  Q  M  H  W  Y  R  A  T  H  Q  E

550       560       570       580       590       600
GCCATCAACTGCTATGCCAATGAGACCTGCTGTGACTTCTTCACGAACCAAGCCTATGCC
CGGTAGTTGACGATACGGTTACTCTGGACGACACTGAAGAAGTGCTTGGTTCGGATACGG
A  I  N  C  Y  A  N  E  T  C  C  D  F  F  T  N  Q  A  Y  A

610       620       630       640       650       660
ATTGCCTCTTCCATCGTGTCCTTCTACGTTCCCCTGGTGATCATGGTCTTCGTCTACTCC
TAACGGAGAAGGTAGCACAGGAAGATGCAAGGGGACCACTAGTACCAGAAGCAGATGAGG
I  A  S  S  I  V  S  F  Y  V  P  L  V  I  M  V  F  V  Y  S

670       680       690       700       710       720
AGGGTCTTTCAGGAGGCCAAAAGGCAGCTCCAGAAGATTGACAAATCTGAGGGCCGCTTC
TCCCAGAAAGTCCTCCGGTTTTCCGTCGAGGTCTTCTAACTGTTTAGACTCCCGGCGAAG
R  V  F  Q  E  A  K  R  Q  L  Q  K  I  D  K  S  E  G  R  F

730       740       750       760       770       780
CATGTCCAGAACCTTAGCCAGGTGGAGCAGGATGGGCGGACGGGGCATGGACTCCGCAGA
GTACAGGTCTTGGAATCGGTCCACCTCGTCCTACCCGCCTGCCCCGTACCTGAGGCGTCT
H  V  Q  N  L  S  Q  V  E  Q  D  G  R  T  G  H  G  L  R  R

790       800       810       820       830       840
TCTTCCAAGTTCTGCTTGAAGGAGCACAAAGCCCTCAAGACGTTAGGCATCATCATGGGC
AGAAGGTTCAAGACGAACTTCCTCGTGTTTCGGGAGTTCTGCAATCCGTAGTAGTACCCG
S  S  K  F  C  L  K  E  H  K  A  L  K  T  L  G  I  I  M  G

850       860       870       880       890       900
ACTTTCACCCTCTGCTGGCTGCCCTTCTTCATCGTTAACATTGTGCATGTGATCCAGGAT
TGAAAGTGGGAGACGACCGACGGGAAGAAGTAGCAATTGTAACACGTACACTAGGTCCTA
T  F  T  L  C  W  L  P  F  F  I  V  N  I  V  H  V  I  Q  D

910       920       930       940       950       960
AACCTCATCCGTAAGGAAGTTTACATCCTCCTAAATTGGATAGGCTATGTCAATTCTGGT
TTGGAGTAGGCATTCCTTCAAATGTAGGAGGATTTAACCTATCCGATACAGTTAAGACCA
N  L  I  R  K  E  V  Y  I  L  L  N  W  I  G  Y  V  N  S  G

970       980       990      1000      1010      1020
TTCAATCCCCTTATCTACTGCCGGAGCCCAGATTTCAGGATTGCCTTCCAGGAGCTTCTG
AAGTTAGGGGAATAGATGACGGCCTCGGGTCTAAAGTCCTAACGGAAGGTCCTCGAAGAC
F  N  P  L  I  Y  C  R  S  P  D  F  R  I  A  F  Q  E  L  L

1030      1040      1050      1060      1070      1080
TGCCTGCGCAGGTCTTCTTTGAAGGCCTATGGGAATGGCTACTCCAGCAACGGCAACACA
ACGGACGCGTCCAGAAGAAACTTCCGGATACCCTTACCGATGAGGTCGTTGCCGTTGTGT
C  L  R  R  S  S  L  K  A  Y  G  N  G  Y  S  S  N  G  N  T

1090      1100      1110      1120      1130      1140
GGGGAGCAGAGTGGATATCACGTGGAACAGGAGAAAGAAAATAAACTGCTGTGTGAAGAC
CCCCTCGTCTCACCTATAGTGCACCTTGTCCTCTTTCTTTTATTTGACGACACACTTCTG
G  E  Q  S  G  Y  H  V  E  Q  E  K  E  N  K  L  L  C  E  D

1150      1160      1170      1180      1190      1200
CTCCCAGGCACGGAAGACTTTGTGGGCCATCAAGGTACTGTGCCTAGCGATAACATTGAT
GAGGGTCCGTGCCTTCTGAAACACCCGGTAGTTCCATGACACGGATCGCTATTGTAACTA
L  P  G  T  E  D  F  V  G  H  Q  G  T  V  P  S  D  N  I  D

1210      1220      1230      1240      1250      1260
TCACAAGGGAGGAATTGTAGTACAAATGACTCACTGCTGGTCGACGGCGGCGGAGGATCT
AGTGTTCCCTCCTTAACATCATGTTTACTGAGTGACGACCAGCTGCCGCCGCCTCCTAGA
S  Q  G  R  N  C  S  T  N  D  S  L  L  V  D  G  G  G  G  S

1270      1280      1290      1300      1310      1320
GGGGGCGGAGGAAGTGGGGGAGGGGGCTCTCTCGAGGACCTGCCTGATGATCACTATCTG
CCCCCGCCTCCTTCACCCCCTCCCCCGAGAGAGCTCCTGGACGGACTACTAGTGATAGAC
G  G  G  G  S  G  G  G  G  S  L  E  D  L  P  D  D  H  Y  L
```

```
1330      1340      1350      1360
TCTACACAGACAATCCTGTCTAAGGATCTGAATGGGACCGACGTGTAA
AGATGTGTCTGTTAGGACAGATTCCTAGACTTACCCTGGCTGCACATT
S  T  Q  T  I  L  S  K  D  L  N  G  T  D  V  *
```

G. Sequence of B2AR-NUB

```
       10        20        30        40        50        60
ATGGGGCAACCCGGGAACGGCAGCGCCTTCTTGCTGGCACCCAATGGAAGCCATGCGCCG
TACCCCGTTGGGCCCTTGCCGTCGCGGAAGAACGACCGTGGGTTACCTTCGGTACGCGGC
M  G  Q  P  G  N  G  S  A  F  L  L  A  P  N  G  S  H  A  P

       70        80        90       100       110       120
GACCACGACGTCACGCAGGAAAGGGACGAGGTGTGGGTGGTGGGCATGGGCATCGTCATG
CTGGTGCTGCAGTGCGTCCTTTCCCTGCTCCACACCCACCACCCGTACCCGTAGCAGTAC
D  H  D  V  T  Q  E  R  D  E  V  W  V  V  G  M  G  I  V  M

      130       140       150       160       170       180
TCTCTCATCGTCCTGGCCATCGTGTTTGGCAATGTGCTGGTCATCACAGCCATTGCCAAG
AGAGAGTAGCAGGACCGGTAGCACAAACCGTTACACGACCAGTAGTGTCGGTAACGGTTC
S  L  I  V  L  A  I  V  F  G  N  V  L  V  I  T  A  I  A  K

      190       200       210       220       230       240
TTCGAGCGTCTGCAGACGGTCACCAACTACTTCATCACTTCACTGGCCTGTGCTGATCTG
AAGCTCGCAGACGTCTGCCAGTGGTTGATGAAGTAGTGAAGTGACCGGACACGACTAGAC
F  E  R  L  Q  T  V  T  N  Y  F  I  T  S  L  A  C  A  D  L

      250       260       270       280       290       300
GTCATGGGCCTGGCAGTGGTGCCCTTTGGGGCCGCCCATATTCTTATGAAAATGTGGACT
CAGTACCCGGACCGTCACCACGGGAAACCCCGGCGGGTATAAGAATACTTTTACACCTGA
V  M  G  L  A  V  V  P  F  G  A  A  H  I  L  M  K  M  W  T

      310       320       330       340       350       360
TTTGGCAACTTCTGGTGCGAGTTTTGGACTTCCATTGATGTGCTGTGCGTCACGGCCAGC
AAACCGTTGAAGACCACGCTCAAAACCTGAAGGTAACTACACGACACGCAGTGCCGGTCG
F  G  N  F  W  C  E  F  W  T  S  I  D  V  L  C  V  T  A  S

      370       380       390       400       410       420
ATTGAGACCCTGTGCGTGATCGCAGTGGATCGCTACTTTGCCATTACTTCACCTTTCAAG
TAACTCTGGGACACGCACTAGCGTCACCTAGCGATGAAACGGTAATGAAGTGGAAAGTTC
I  E  T  L  C  V  I  A  V  D  R  Y  F  A  I  T  S  P  F  K

      430       440       450       460       470       480
TACCAGAGCCTGCTGACCAAGAATAAGGCCCGGGTGATCATTCTGATGGTGTGGATTGTG
ATGGTCTCGGACGACTGGTTCTTATTCCGGGCCCACTAGTAAGACTACCACACCTAACAC
Y  Q  S  L  L  T  K  N  K  A  R  V  I  I  L  M  V  W  I  V

      490       500       510       520       530       540
TCAGGCCTTACCTCCTTCTTGCCCATTCAGATGCACTGGTACCGGGCCACCCACCAGGAA
AGTCCGGAATGGAGGAAGAACGGGTAAGTCTACGTGACCATGGCCCGGTGGGTGGTCCTT
S  G  L  T  S  F  L  P  I  Q  M  H  W  Y  R  A  T  H  Q  E

      550       560       570       580       590       600
GCCATCAACTGCTATGCCAATGAGACCTGCTGTGACTTCTTCACGAACCAAGCCTATGCC
CGGTAGTTGACGATACGGTTACTCTGGACGACACTGAAGAAGTGCTTGGTTCGGATACGG
A  I  N  C  Y  A  N  E  T  C  C  D  F  F  T  N  Q  A  Y  A

      610       620       630       640       650       660
ATTGCCTCTTCCATCGTGTCCTTCTACGTTCCCCTGGTGATCATGGTCTTCGTCTACTCC
TAACGGAGAAGGTAGCACAGGAAGATGCAAGGGGACCACTAGTACCAGAAGCAGATGAGG
I  A  S  S  I  V  S  F  Y  V  P  L  V  I  M  V  F  V  Y  S

      670       680       690       700       710       720
AGGGTCTTTCAGGAGGCCAAAAGGCAGCTCCAGAAGATTGACAAATCTGAGGGCCGCTTC
TCCCAGAAAGTCCTCCGGTTTTCCGTCGAGGTCTTCTAACTGTTTAGACTCCCGGCGAAG
R  V  F  Q  E  A  K  R  Q  L  Q  K  I  D  K  S  E  G  R  F

      730       740       750       760       770       780
CATGTCCAGAACCTTAGCCAGGTGGAGCAGGATGGGCGGACGGGGCATGGACTCCGCAGA
GTACAGGTCTTGGAATCGGTCCACCTCGTCCTACCCGCCTGCCCCGTACCTGAGGCGTCT
H  V  Q  N  L  S  Q  V  E  Q  D  G  R  T  G  H  G  L  R  R

      790       800       810       820       830       840
TCTTCCAAGTTCTGCTTGAAGGAGCACAAAGCCCTCAAGACGTTAGGCATCATCATGGGC
AGAAGGTTCAAGACGAACTTCCTCGTGTTTCGGGAGTTCTGCAATCCGTAGTAGTACCCG
S  S  K  F  C  L  K  E  H  K  A  L  K  T  L  G  I  I  M  G
```

```
        850       860       870       880       890       900
ACTTTCACCCTCTGCTGGCTGCCCTTCTTCATCGTTAACATTGTGCATGTGATCCAGGAT
TGAAAGTGGGAGACGACCGACGGGAAGAAGTAGCAATTGTAACACGTACACTAGGTCCTA
T  F  T  L  C  W  L  P  F  F  I  V  N  I  V  H  V  I  Q  D

        910       920       930       940       950       960
AACCTCATCCGTAAGGAAGTTTACATCCTCCTAAATTGGATAGGCTATGTCAATTCTGGT
TTGGAGTAGGCATTCCTTCAAATGTAGGAGGATTTAACCTATCCGATACAGTTAAGACCA
N  L  I  R  K  E  V  Y  I  L  L  N  W  I  G  Y  V  N  S  G

        970       980       990      1000      1010      1020
TTCAATCCCCTTATCTACTGCCGGAGCCCAGATTTCAGGATTGCCTTCCAGGAGCTTCTG
AAGTTAGGGGAATAGATGACGGCCTCGGGTCTAAAGTCCTAACGGAAGGTCCTCGAAGAC
F  N  P  L  I  Y  C  R  S  P  D  F  R  I  A  F  Q  E  L  L

       1030      1040      1050      1060      1070      1080
TGCCTGCGCAGGTCTTCTTTGAAGGCCTATGGGAATGGCTACTCCAGCAACGGCAACACA
ACGGACGCGTCCAGAAGAAACTTCCGGATACCCTTACCGATGAGGTCGTTGCCGTTGTGT
C  L  R  R  S  S  L  K  A  Y  G  N  G  Y  S  S  N  G  N  T

       1090      1100      1110      1120      1130      1140
GGGGAGCAGAGTGGATATCACGTGGAACAGGAGAAAGAAAATAAACTGCTGTGTGAAGAC
CCCCTCGTCTCACCTATAGTGCACCTTGTCCTCTTTCTTTTATTTGACGACACACTTCTG
G  E  Q  S  G  Y  H  V  E  Q  E  K  E  N  K  L  L  C  E  D

       1150      1160      1170      1180      1190      1200
CTCCCAGGCACGGAAGACTTTGTGGGCCATCAAGGTACTGTGCCTAGCGATAACATTGAT
GAGGGTCCGTGCCTTCTGAAACACCCGGTAGTTCCATGACACGGATCGCTATTGTAACTA
L  P  G  T  E  D  F  V  G  H  Q  G  T  V  P  S  D  N  I  D

       1210      1220      1230      1240      1250      1260
TCACAAGGGAGGAATTGTAGTACAAATGACTCACTGCTGTACCCAGCTTTCTTGTACAAA
AGTGTTCCCTCCTTAACATCATGTTTACTGAGTGACGACATGGGTCGAAAGAACATGTTT
S  Q  G  R  N  C  S  T  N  D  S  L  L  Y  P  A  F  L  Y  K

       1270      1280      1290      1300      1310      1320
GTGGTTGATTCTAGAGGTGGCGGTGGCTCTGGAGGTGGTGGGTCCGACTACAAAGACCAT
CACCAACTAAGATCTCCACCGCCACCGAGACCTCCACCACCCAGGCTGATGTTTCTGGTA
V  V  D  S  R  G  G  G  G  S  G  G  G  G  S  D  Y  K  D  H

       1330      1340      1350      1360      1370      1380
GACGGTGATTATAAAGATCATGACATCGATTACAAGGATGACGATGACAAGATGCAGATT
CTGCCACTAATATTTCTAGTACTGTAGCTAATGTTCCTACTGCTACTGTTCTACGTCTAA
D  G  D  Y  K  D  H  D  I  D  Y  K  D  D  D  D  K  M  Q  I

       1390      1400      1410      1420      1430      1440
TTCGTCAAGACTTTGACCGGTAAAACCATAACATTGGAAGTTGAATCTTCCGATACCATC
AAGCAGTTCTGAAACTGGCCATTTTGGTATTGTAACCTTCAACTTAGAAGGCTATGGTAG
F  V  K  T  L  T  G  K  T  I  T  L  E  V  E  S  S  D  T  I

       1450      1460      1470      1480
GACAACGTTAAGTCGAAAATTCAAGACAAGGAAGGTATCTAA
CTGTTGCAATTCAGCTTTTAAGTTCTGTTCCTTCCATAGATT
D  N  V  K  S  K  I  Q  D  K  E  G  I  *
```

H. Sequence of CD25-CUB-TF

```
        10        20        30        40        50        60
ATGGATTCATACCTGCTGATGTGGGGACTGCTCACGTTCATCATGGTGCCTGGCTGCCAG
TACCTAAGTATGGACGACTACACCCCTGACGAGTGCAAGTAGTACCACGGACCGACGGTC
 M  D  S  Y  L  L  M  W  G  L  L  T  F  I  M  V  P  G  C  Q

        70        80        90       100       110       120
GCAGAACTCTGTGACGATGACCCGCCAGAGATCCCACACGCCACATTCAAAGCCATGGCC
CGTCTTGAGACACTGCTACTGGGCGGTCTCTAGGGTGTGCGGTGTAAGTTTCGGTACCGG
 A  E  L  C  D  D  D  P  P  E  I  P  H  A  T  F  K  A  M  A

       130       140       150       160       170       180
TACAAGGAAGGAACCATGTTGAACTGTGAATGCAAGAGAGGTTTCCGCAGAATAAAAAGC
ATGTTCCTTCCTTGGTACAACTTGACACTTACGTTCTCTCCAAAGGCGTCTTATTTTTCG
 Y  K  E  G  T  M  L  N  C  E  C  K  R  G  F  R  R  I  K  S
```

```
        190       200       210       220       230       240
GGGTCACTCTATATGCTCTGTACAGGAAACTCTAGCCACTCGTCCTGGGACAACCAATGT
CCCAGTGAGATATACGAGACATGTCCTTTGAGATCGGTGAGCAGGACCCTGTTGGTTACA
 G  S  L  Y  M  L  C  T  G  N  S  S  H  S  S  W  D  N  Q  C

        250       260       270       280       290       300
CAATGCACAAGCTCTGCCACTCGGAACACAACGAAACAAGTGACACCTCAACCTGAAGAA
GTTACGTGTTCGAGACGGTGAGCCTTGTGTTGCTTTGTTCACTGTGGAGTTGGACTTCTT
 Q  C  T  S  S  A  T  R  N  T  T  K  Q  V  T  P  Q  P  E  E

        310       320       330       340       350       360
CAGAAAGAAAGGAAAACCACAGAAATGCAAAGTCCAATGCAGCCAGTGGACCAAGCGAGC
GTCTTTCTTTCCTTTTGGTGTCTTTACGTTTCAGGTTACGTCGGTCACCTGGTTCGCTCG
 Q  K  E  R  K  T  T  E  M  Q  S  P  M  Q  P  V  D  Q  A  S

        370       380       390       400       410       420
CTTCCAGGTCACTGCAGGGAACCTCCACCATGGGAAAATGAAGCCACAGAGAGAATTTAT
GAAGGTCCAGTGACGTCCCTTGGAGGTGGTACCCTTTTACTTCGGTGTCTCTCTTAAATA
 L  P  G  H  C  R  E  P  P  P  W  E  N  E  A  T  E  R  I  Y

        430       440       450       460       470       480
CATTTCGTGGTGGGGCAGATGGTTTATTATCAGTGCGTCCAGGGATACAGGGCTCTACAC
GTAAAGCACCACCCCGTCTACCAAATAATAGTCACGCAGGTCCCTATGTCCCGAGATGTG
 H  F  V  V  G  Q  M  V  Y  Y  Q  C  V  Q  G  Y  R  A  L  H

        490       500       510       520       530       540
AGAGGTCCTGCTGAGAGCGTCTGCAAAATGACCCACGGGAAGACAAGGTGGACCCAGCCC
TCTCCAGGACGACTCTCGCAGACGTTTTACTGGGTGCCCTTCTGTTCCACCTGGGTCGGG
 R  G  P  A  E  S  V  C  K  M  T  H  G  K  T  R  W  T  Q  P

        550       560       570       580       590       600
CAGCTCATATGCACAGGTGAAATGGAGACCAGTCAGTTTCCAGGTGAAGAGAAGCCTCAG
GTCGAGTATACGTGTCCACTTTACCTCTGGTCAGTCAAAGGTCCACTTCTCTTCGGAGTC
 Q  L  I  C  T  G  E  M  E  T  S  Q  F  P  G  E  E  K  P  Q

        610       620       630       640       650       660
GCAAGCCCCGAAGGCCGTCCTGAGAGTGAGACTTCCTGCCTCGTCACAACAACAGATTTT
CGTTCGGGGCTTCCGGCAGGACTCTCACTCTGAAGGACGGAGCAGTGTTGTTGTCTAAAA
 A  S  P  E  G  R  P  E  S  E  T  S  C  L  V  T  T  T  D  F

        670       680       690       700       710       720
CAAATACAGACAGAAATGGCTGCAACCATGGAGACGTCCATATTTACAACAGAGTACCAG
GTTTATGTCTGTCTTTACCGACGTTGGTACCTCTGCAGGTATAAATGTTGTCTCATGGTC
 Q  I  Q  T  E  M  A  A  T  M  E  T  S  I  F  T  T  E  Y  Q

        730       740       750       760       770       780
GTAGCAGTGGCCGGCTGTGTTTTCCTGCTGATCAGCGTCCTCCTCCTGAGTGGGCTCACC
CATCGTCACCGGCCGACACAAAAGGACGACTAGTCGCAGGAGGAGGACTCACCCGAGTGG
 V  A  V  A  G  C  V  F  L  L  I  S  V  L  L  L  S  G  L  T

        790       800       810       820       830       840
TGGCAGCGGAGACAGAGGAAGAGTAGAAGAACAATCTACCCAGCTTTCTTGTACAAAGTG
ACCGTCGCCTCTGTCTCCTTCTCATCTTCTTGTTAGATGGGTCGAAAGAACATGTTTCAC
 W  Q  R  R  Q  R  K  S  R  R  T  I  Y  P  A  F  L  Y  K  V

        850       860       870       880       890       900
GTTGATTCTAGAGGTGGCGGTGGCTCTGGAGGTGGTGGGTCCATGTCGGGGGGGATCCCT
CAACTAAGATCTCCACCGCCACCGAGACCTCCACCACCCAGGTACAGCCCCCCCTAGGGA
 V  D  S  R  G  G  G  G  S  G  G  G  G  S  M  S  G  G  I  P

        910       920       930       940       950       960
CCAGATCAACAAAGATTGATCTTTGCCGGTAAGCAGCTAGAAGACGGTAGAACGCTGTCT
GGTCTAGTTGTTTCTAACTAGAAACGGCCATTCGTCGATCTTCTGCCATCTTGCGACAGA
 P  D  Q  Q  R  L  I  F  A  G  K  Q  L  E  D  G  R  T  L  S

        970       980       990      1000      1010      1020
GATTACAACATTCAGAAGGAGTCCACCTTACATCTTGTGCTAAGGCTAAGAGGTGGTATG
CTAATGTTGTAAGTCTTCCTCAGGTGGAATGTAGAACACGATTCCGATTCTCCACCATAC
 D  Y  N  I  Q  K  E  S  T  L  H  L  V  L  R  L  R  G  G  M

       1030      1040      1050      1060      1070      1080
CACAGATCAGCTTTGTCGACGGTATCGATAAGCTTGATGAAGCTACTGTCTTCTATCGAA
GTGTCTAGTCGAAACAGCTGCCATAGCTATTCGAACTACTTCGATGACAGAAGATAGCTT
 H  R  S  A  L  S  T  V  S  I  S  L  M  K  L  L  S  S  I  E

       1090      1100      1110      1120      1130      1140
CAAGCATGCGATATTTGCCGACTTAAAAAGCTCAAGTGCTCCAAAGAAAAACCGAAGTGC
GTTCGTACGCTATAAACGGCTGAATTTTTCGAGTTCACGAGGTTTCTTTTTGGCTTCACG
 Q  A  C  D  I  C  R  L  K  K  L  K  C  S  K  E  K  P  K  C
```

```
        1150        1160        1170        1180        1190        1200
GCCAAGTGTCTGAAGAACAACTGGGAGTGTCGCTACTCTCCCAAAACCAAAAGGTCTCCG
CGGTTCACAGACTTCTTGTTGACCCTCACAGCGATGAGAGGGTTTTGGTTTTCCAGAGGC
 A  K  C  L  K  N  N  W  E  C  R  Y  S  P  K  T  K  R  S  P

        1210        1220        1230        1240        1250        1260
CTGACTAGGGCACATCTGACAGAAGTGGAATCAAGGCTAGAAAGACTGGAACAGCTATTT
GACTGATCCCGTGTAGACTGTCTTCACCTTAGTTCCGATCTTTCTGACCTTGTCGATAAA
 L  T  R  A  H  L  T  E  V  E  S  R  L  E  R  L  E  Q  L  F

        1270        1280        1290        1300        1310        1320
CTACTGATTTTTCCTCGAGAAGACCTTGACATGATTTTGAAAATGGATTCTTTACAGGAT
GATGACTAAAAAGGAGCTCTTCTGGAACTGTACTAAAACTTTTACCTAAGAAATGTCCTA
 L  L  I  F  P  R  E  D  L  D  M  I  L  K  M  D  S  L  Q  D

        1330        1340        1350        1360        1370        1380
ATAAAAGCATTGTTAACAGGATTATTTGTACAAGATAATGTGAATAAAGATGCCGTCACA
TATTTTCGTAACAATTGTCCTAATAAACATGTTCTATTACACTTATTTCTACGGCAGTGT
 I  K  A  L  L  T  G  L  F  V  Q  D  N  V  N  K  D  A  V  T

        1390        1400        1410        1420        1430        1440
GATAGATTGGCTTCAGTGGAGACTGATATGCCTCTAACATTGAGACAGCATAGAATAAGT
CTATCTAACCGAAGTCACCTCTGACTATACGGAGATTGTAACTCTGTCGTATCTTATTCA
 D  R  L  A  S  V  E  T  D  M  P  L  T  L  R  Q  H  R  I  S

        1450        1460        1470        1480        1490        1500
GCGACATCATCATCGGAAGAGAGTAGTAACAAAGGTCAAAGACAGTTGACTGTATCGATT
CGCTGTAGTAGTAGCCTTCTCTCATCATTGTTTCCAGTTTCTGTCAACTGACATAGCTAA
 A  T  S  S  S  E  E  S  S  N  K  G  Q  R  Q  L  T  V  S  I

        1510        1520        1530        1540        1550        1560
TTTTCCCCCATGCTGTTACCATCAGGGCAGATCTCAAACCAGGCCCTGGCCTTAGCACCG
AAAAGGGGGTACGACAATGGTAGTCCCGTCTAGAGTTTGGTCCGGGACCGGAATCGTGGC
 F  S  P  M  L  L  P  S  G  Q  I  S  N  Q  A  L  A  L  A  P

        1570        1580        1590        1600        1610        1620
TCCTCTGCCCCAGTCCTTGCCCAGACCATGGTCCCTTCCTCAGCCATGGTACCTCTGGCT
AGGAGACGGGGTCAGGAACGGGTCTGGTACCAGGGAAGGAGTCGGTACCATGGAGACCGA
 S  S  A  P  V  L  A  Q  T  M  V  P  S  S  A  M  V  P  L  A

        1630        1640        1650        1660        1670        1680
CAGCCCCCAGCTCCTGCCCCAGTTCTAACCCCGGGTCCTCCCCAGTCCCTGTCTGCACCT
GTCGGGGGTCGAGGACGGGGTCAAGATTGGGGCCCAGGAGGGGTCAGGGACAGACGTGGA
 Q  P  P  A  P  A  P  V  L  T  P  G  P  P  Q  S  L  S  A  P

        1690        1700        1710        1720        1730        1740
GTTCCAAAGAGCACCCAGGCTGGGGAAGGCACGCTGTCGGAAGCCCTGCTGCACCTGCAG
CAAGGTTTCTCGTGGGTCCGACCCCTTCCGTGCGACAGCCTTCGGGACGACGTGGACGTC
 V  P  K  S  T  Q  A  G  E  G  T  L  S  E  A  L  L  H  L  Q

        1750        1760        1770        1780        1790        1800
TTTGATGCTGATGAAGACTTGGGGGCCTTGCTTGGCAACAGCACAGACCCAGGAGTGTTC
AAACTACGACTACTTCTGAACCCCCGGAACGAACCGTTGTCGTGTCTGGGTCCTCACAAG
 F  D  A  D  E  D  L  G  A  L  L  G  N  S  T  D  P  G  V  F

        1810        1820        1830        1840        1850        1860
ACAGACCTGGCATCTGTGGACAACTCAGAGTTTCAGCAGCTCCTGAACCAGGGTGTGTCC
TGTCTGGACCGTAGACACCTGTTGAGTCTCAAAGTCGTCGAGGACTTGGTCCCACACAGG
 T  D  L  A  S  V  D  N  S  E  F  Q  Q  L  L  N  Q  G  V  S

        1870        1880        1890        1900        1910        1920
ATGTCTCACTCCACAGCTGAGCCCATGCTGATGGAGTACCCTGAAGCTATAACTCGCCTG
TACAGAGTGAGGTGTCGACTCGGGTACGACTACCTCATGGGACTTCGATATTGAGCGGAC
 M  S  H  S  T  A  E  P  M  L  M  E  Y  P  E  A  I  T  R  L

        1930        1940        1950        1960        1970        1980
GTGACAGGGTCCCAGAGGCCCCCTGACCCAGCTCCCACACCCCTGGGGACCTCGGGGCTT
CACTGTCCCAGGGTCTCCGGGGGACTGGGTCGAGGGTGTGGGGACCCCTGGAGCCCCGAA
 V  T  G  S  Q  R  P  P  D  P  A  P  T  P  L  G  T  S  G  L

        1990        2000        2010        2020        2030        2040
CCCAATGGTCTCTCCGGAGATGAAGACTTCTCCTCCATTGCGGACATGGACTTCTCTGCT
GGGTTACCAGAGAGGCCTCTACTTCTGAAGAGGAGGTAACGCCTGTACCTGAAGAGACGA
 P  N  G  L  S  G  D  E  D  F  S  S  I  A  D  M  D  F  S  A
```

```
        2050      2060      2070      2080      2090      2100
CTTTTGAGTCAGATCAGCTCCGGAGGTGGCGGAGGTAAGCCTATCCCTAACCCTCTCCTC
GAAAACTCAGTCTAGTCGAGGCCTCCACCGCCTCCATTCGGATAGGGATTGGGAGAGGAG
 L  L  S  Q  I  S  S  G  G  G  G  G  K  P  I  P  N  P  L  L

        2110
GGTCTCGATTCTACGTAA
CCAGAGCTAAGATGCATT
 G  L  D  S  T  *
```

Supplementary Table S1

A

Thymocyte population	Markers used in thymocyte fractionation	*LMO2* TPM	*CD34* TPM	*CD4* TPM	*CD8A* TPM	*CD8B TPM*
Thy1.1	CD34+CD7-CD1a-CD4-CD8-	87.8	307.5	82.8	5.6	0.4
Thy1.2	CD34+CD7-CD1a-CD4-CD8-	295.8	1392.9	0	10.7	10.5
Thy2.1	CD34+CD7+CD1a-CD4-CD8-	53.0	404.8	0	0	0.8
Thy2.2	CD34+CD7+CD1a-CD4-CD8-	22.2	369.5	0.8	0.6	0
Thy3.1	CD34+CD7+CD1a+CD4-CD8-	3.1	73.4	9.5	1.6	2.8
Thy3.2	CD34+CD7+CD1a+CD4-CD8-	1.1	76.9	12.7	1.1	2.9
Thy4.1	CD4+CD8+	0.5	0.8	393.3	254.4	168.8
Thy4.2	CD4+CD8+	0	0	611.2	1006.3	659.4
Thy5.1	CD3+CD4+CD8-	0	1.0	291.0	9.3	14.7
Thy5.2	CD3+CD4+CD8-	1.3	0	371.8	44.4	22.0
Thy6.1	CD3+CD4-CD8+	1.4	1.2	14.6	667.5	208.2
Thy6.2	CD3+CD4-CD8+	0	0	5.3	859.4	398.8

B

Mouse number	DN1	DN2	DN3	DN4
1	0.05	0.4	0.1	0.04
2	2.1	0.5	0.03	0.05
3	1.0	0.7	0.1	0.03

Supplementary Table S1 Expression of LMO2 mRNA in normal human and mouse thymocytes compared to surface markers used in fractionation of thymus populations

Panel A. Data are shown as TPM for each of the duplicate human thymocyte populations from Casero, Sandoval, Seet, Zhu, Ha, Luong, Parekh, Crooks Nature Immunology 16, 1282-91, 2015 doi: 10.1038/ni.3299

Panel B. Summary of data LMO2 TPM data from P19-21 wild type mouse fractionated thymocytes

Supplementary Table S2

Surfaceome expression levels in Lck-Lmo2 transgenic and wild type DN cells. Each sheet on the table contains the average TPM expression levels in one of the DN stages for wild type and Lck-Lmo2 samples (in columns F and G respectively). Columns H and I contain the expression class, as defined in Figure 1 (0 means TPM <0.1, LOW means TPM between 0.1 and 1, MED means TPM between 1 and 10 and HIGH means TPM higher than 10).

https://www.rdm.ox.ac.uk/about/our-divisions/nuffield-division-of-clinical-laboratory-sciences/nuffield-division-of-clinical-laboratory-sciences-research/rabbitts-group/rabbitts-group-news/supplementary-table-s1

Supplementary Table S3

A.

DN1 high;low	DN2 high; 0	DN2 high; low	DN3 high; 0	DN3 high; low	DN4 high; 0	DN4 high; low
Angpt1	Atp8a2	Lmcd1	Efemp2	F2r	Atp8a2	Angpt1
Gpr56		Ly6e	Lalba	Psma6	Insig2	Bpifc
Ifitm1		Serpine2	Lmcd1	Serpine2	Lalba	Cd59a
Ly6c1		Tspan32		Tspan32	Lmcd1	F2r
						Lcn4
						Mzb1
						Scin

B.

Gene name	Genecard URL	Surface or secreted
Angpt1	http://www.genecards.org/cgi-bin/carddisp.pl?gene=ANGPT1	Secreted
Gpr56	http://www.genecards.org/cgi-bin/carddisp.pl?gene=ADGRG1	Surface
CD225	http://www.genecards.org/cgi-bin/carddisp.pl?gene=IFITM1	Surface
Ly6c1	n/a	Surface
Atp8a2	http://www.genecards.org/cgi-bin/carddisp.pl?gene=Atp8a2	Surface
Lmcd1	http://www.genecards.org/cgi-bin/carddisp.pl?gene=LMCD1	nk
Tspan32	http://www.genecards.org/cgi-bin/carddisp.pl?gene=TSPAN32	Surface
Serpine2	http://www.genecards.org/cgi-bin/carddisp.pl?gene=SERPINE2	Secreted
Ly6e	http://www.genecards.org/cgi-bin/carddisp.pl?gene=LY6E	Surface
Lalba	http://www.genecards.org/cgi-bin/carddisp.pl?gene=LALBA	Secreted
Efemp2	http://www.genecards.org/cgi-bin/carddisp.pl?gene=EFEMP2	Secreted
Psma6	http://www.genecards.org/cgi-bin/carddisp.pl?gene=PSMA6	nk
F2r	http://www.genecards.org/cgi-bin/carddisp.pl?gene=F2R	Surface
Insig2	http://www.genecards.org/cgi-bin/carddisp.pl?gene=INSIG2	ER
CD53	http://www.genecards.org/cgi-bin/carddisp.pl?gene=CD53	Surface
CD59	http://www.genecards.org/cgi-bin/carddisp.pl?gene=CD59	Surface
CD7	http://www.genecards.org/cgi-bin/carddisp.pl?gene=CD7	Surface

C.

DN1 high; 0	DN2 high; low	DN3 high; low	DN4 high; low
GRP56	GPR56	GPR56	GPR56
	CD53	CD53	CD53
CD59a	CD59a	CD59a	CD59a

Supplementary Table S3. Candidate mRNAs differentially expressed between age matched *Lck-Lmo2* transgenic and wild type DN cells.

A. Each column shows the gene name encoding the mRNA found in the RNA-seq surfaceome analysis where the expression in the *Lck-Lmo2* mRNA is high vs low or high vs 0 expression as indicated.

One mRNA (*Atp8a2*) is expressed in *Lck-Lmo2* DN2 and absent from wild type DN2 and four mRNAs (*Lmcd1, Ly6e, Serpine2* and *Tspan32*) have higher expression in *Lck-Lmo2* DN2 than in wild type. Similarly, three mRNAs (*Efemp2, Lalba* and *Lmcd1*) are expressed in *Lck-Lmo2* DN3 and absent in wild type DN3 and four mRNAs (*F2r, Psma6, Serpine2* and *Tspan32*) have higher expression in *Lck-Lmo2* DN3 than in wild type. From this restricted set of mRNAs, several encode proteins that are surface-

associated and others are secreted (Figure 1J). Of particular interest is *Tspan32* (a member of the tetraspanin family) that encodes a protein found in haematopoiesis and that has been implicated in malignancy.

B. Gene card information mRNAs differentially expressed between *Lck-Lmo2* transgenic and wild type DN cells.

The table lists the gene card information for each gene encoding mRNAs identified from the RNA-seq data. The Genecard URLs are listed and whether the protein products are found in the cell surface or secreted.

nk= not known

Insig2 is an endoplasmic reticulum (ER) associated protein

C. Each column shows the gene name encoding the mRNA found in the RNA-seq surfaceome analysis where the expression in the *Lck-Lmo2* mRNA is at least 10 x higher than the wild-type equivalents

Supplementary Table S4

TPM values for mRNAs that are exclusive to DN subsets (extracted from the RNA–seq data) or common to groups of DN subsets as indicated for development of the Venn diagram in Figure 1 (A-B)

https://www.rdm.ox.ac.uk/about/our-divisions/nuffield-division-of-clinical-laboratory-sciences/nuffield-division-of-clinical-laboratory-sciences-research/rabbitts-group/rabbitts-group-news/supplementary-table-s2

Supplementary Table S5

TPM values for all mRNAs extracted from the RNA-seq data for development of the Venn diagrams in Figure 1 (A-B).

https://www.rdm.ox.ac.uk/about/our-divisions/nuffield-division-of-clinical-laboratory-sciences/nuffield-division-of-clinical-laboratory-sciences-research/rabbitts-group/rabbitts-group-news/supplementary-table-s3

Supplementary Table S6

Human T-ALL primary	Translocation	TPM value for LMO2
T1	t(11;14)(p13;q11)	35.37
T2	t(11;14)(p13;q11)	94.16
T3	t(11;14)(p13;q11)	
T4	t(11;14)(p13;q11)	
NT1	n/a	2.89
NT2	n/a	4.50
NT3	n/a	7.77
NT4	n/a	4.50
NT5	n/a	nd
NT6	n/a	nd
NT7	n/a	nd
NT8	n/a	nd
NT9	n/a	nd
NT10	n/a	nd
NT11	n/a	nd

Human T-ALL samples used in this study.
T- *LMO2* translocation present
NT- no *LMO2* translocation present

Supplementary Table S7

Surfaceome TPM expression levels in *Lck-Lmo2* transgenic tumours and human T cell tumours. RNA-seq TPM values for two *Lck-Lmo2* transgenic tumours and six human T cell tumours, two with LMO2-associated chromosomal translocations.

https://www.rdm.ox.ac.uk/about/our-divisions/nuffield-division-of-clinical-laboratory-sciences/nuffield-division-of-clinical-laboratory-sciences-research/rabbitts-group/rabbitts-group-news/supplementary-table-s6

Supplementary Table S8

A. Human T-ALL

mRNA	T1	T2	NT1	NT2	NT3	NT4
CD7	170	123	231	155	101	441
CD53	67	144	91	141	57	121
CD59	52	35	21	26	6	8
GPR56	26	59	24	40	35	17

B. Mouse

mRNA	Ensembl code	Tg DN1	Tg DN2	Tg DN3	Tg DN4	Wt DN1	Wt DN2	Wt DN3	Wt Dn4
CD53	ENSMUST00000038845	284	203	183	326	221	82	27	26
CD59a	ENSMUST00000040423	8	33	26	28	0.2	6	4	1
GPR56	ENSMUST00000093271	13	83	51	42	1	41	14	2
GPR56	ENSMUST00000179619	24	160	107	97	2	58	20	22

C. Mouse Tg/wt TPM ratios

mRNA	DN1	DN2	DN3	DN4
CD53	1.3	3	7	13
CD59a	40	6	7	28
GPR56	13	2	4	21
GPR56	12	3	5	4

Supplementary Table S8. Expression of GPR56, CD53, and CD59a in mouse T cells and primary human T-ALL.

A. mRNA TPM values for *CD53, CD59* and GPR56 in the six human T-ALL samples for which RNA-seq data are available.

B. mRNA TPM values for *Cd53, Cd59* and Gpr56 in the *Lck-Lmo2* and wild types DN cells. Two coding, spice variants of *Gpr56* are shown.

C. The ratio of mRNA expression levels for *Cd53, Cd59* and Gpr56 in the *Lck-Lmo2* and wild types DN cells
